# Selective shaping of prokaryotic communities and core symbiont maintenance suggest large-scale aquarium facilities as reservoirs of microbiome diversity in octocorals

**DOI:** 10.3389/fmicb.2025.1651109

**Published:** 2025-09-03

**Authors:** Matilde Marques, Francisco Pascoal, Helena Villela, Elsa Santos, Núria Baylina, Raquel S. Peixoto, Tina Keller-Costa, Rodrigo Costa

**Affiliations:** ^1^Institute for Bioengineering and Biosciences (iBB) and Institute for Health and Bioeconomy (i4HB), Instituto Superior Técnico (IST), University of Lisbon, Lisbon, Portugal; ^2^Department of Bioengineering, Instituto Superior Técnico (IST), University of Lisbon, Lisbon, Portugal; ^3^Interdisciplinary Centre of Marine and Environmental Research (CIIMAR), University of Porto, Porto, Portugal; ^4^Department of Biology, Faculty of Sciences, University of Porto, Porto, Portugal; ^5^Division of Biological and Environmental Science and Engineering (BESE), King Abdullah University of Science and Technology (KAUST), Thuwal, Saudi Arabia; ^6^Oceanário de Lisboa, Esplanada D. Carlos I, Lisbon, Portugal

**Keywords:** *Litophyton*, mesocosm, *Endozoicomonas*, microbiome stewardship, symbiosis

## Abstract

**Introduction:**

Octocorals play a critical role in coral ecosystems, contributing to habitat complexity and marine biodiversity. Despite their ecological importance, the microbial communities associated with octocorals remain understudied, particularly under ex situ conditions.

**Methods:**

This study compared the prokaryotic communities of the tropical octocoral *Litophyton* sp., surrounding seawater, and sediments (“biotopes”) from a natural Red Sea reef and a long-term tropical aquarium mesocosm designed to emulate natural reef ecosystems (“habitats”). Using high throughput 16S rRNA gene sequencing, we assessed community composition, diversity, and core taxa.

**Results:**

Distinct prokaryotic assemblages were associated with each biotope, with core symbionts persisting across habitats. While seawater communities diverged between habitats, sediment communities were compositionally more similar, dominated by *Nitrosopumilaceae*, *Pirellulaceae*, *Woeseiaceae*, and *Flavobacteriaceae*. *Litophyton* sp. harbored specific symbionts consistently across habitats. Alpha-diversity in *Litophyton* sp. did not differ significantly between habitats (ANOVA with Tukey’s HSD, *p* > 0.05), and beta-diversity patterns were also not significant (PERMANOVA, *p* > 0.05). We identified 19 ASVs shared across *Litophyton* sp. habitats, dominated by *Endozoicomonas*, unclassified *Campylobacterales*, and *Marivibrio*. Several core families, such as *Endozoicomonadaceae*, *Spirochaetaceae*, and *Kiloniellaceae* were consistently associated with *Litophyton* sp. across habitats, indicating stability of specific host-microbe associations even after 25 years in aquarium conditions. Phylogenetic analysis further demonstrated the selective maintenance of diverse *Endozoicomonas* lineages in aquarium-kept *Litophyton* specimens.

**Discussion:**

These findings suggest that large-scale aquarium ecosystems can preserve, to some extent, the structure and diversity of coral-associated microbiomes over extended time periods. By supporting key symbiotic taxa, multi-trophic integrated aquarium systems may serve as repositories for healthy coral-associated microbial communities and microbiome stewardship, underscoring their value in future conservation efforts to sustain the biodiversity of marine holobionts in the face of growing environmental challenges.

## 1 Introduction

Octocorallia, a class within the subphylum Anthozoa (Phylum Cnidaria; Haeckel, 1866), comprises over 3,500 species of corals that are globally distributed across diverse oceanic climates and depths (McFadden et al., 2022). These species enhance habitat complexity and biodiversity in regions where they are abundant (McFadden et al., 2022). The combined effects of climate change—including rising seawater temperatures, oxygen depletion, and ocean acidification, and other anthropogenic-induced disturbances have driven shifts in marine benthic communities, typically towards a dominance of algae, marine sponges or octocorals over the previously prevailing hard corals (hexacorals/Hexacorallia) in several tropical regions (Bell et al., 2022; Sánchez et al., 2019; Tsounis and Edmunds, 2017; van de Water et al., 2018a). However, increasing environmental stress and human activities have led to substantial mortality among octocorals in some regions, particularly in the Mediterranean Sea and Belizean Barrier Reef (Alves et al., 2022; Ponti et al., 2014; Vezzulli et al., 2010).

Together with a range of microorganisms such as microalgae, archaea, viruses, fungi and/or protists that compose the coral microbiota, bacteria have long been recognized as important contributors to coral health (Bourne et al., 2016; Knowlton and Rohwer, 2003; Thurber et al., 2017). Bacteria have been suggested to participate in nitrogen, sulfur and carbon cycling, as well as to provide protection against pathogens, and facilitate acclimatization to environmental changes (Peixoto et al., 2017). Despite their global distribution, diversity, and prevalence across reef ecosystems, the microbiota of octocorals remains understudied compared to that of hexacorals. Current work shows that octocoral bacterial communities are shaped by host phylogeny, depth, season, and regional factors. In tropical species, host-specific assemblages are often dominated by *Endozoicomonas*, *Spirochaeta*, and *Mycoplasma* (Cleary et al., 2021; Jahajeeah et al., 2023; McCauley et al., 2020; Monti et al., 2023; Osman et al., 2020; Park et al., 2022). Temperate octocorals may display more variability in community composition yet still retain the same core taxa, alongside *Alteromonadales*, *Flavobacteriales*, and *Cellvibrionales* (Bonacolta et al., 2024; Haydon et al., 2022; Keller-Costa et al., 2021; Steinum et al., 2024; van de Water et al., 2016). Coldwater octocorals seem to exhibit a more taxonomically conserved microbiome, predominantly featuring *Alteromonadales*, *Oceanospirillales* (including *Endozoicomonas*), *Spirochaetales*, and *Tenericutes*, though research remains scarce (Goldsmith et al., 2018; Kellogg and Pratte, 2021; Vohsen and Herrera, 2024; Weiler et al., 2018).

Shifts in microbiome composition during dysbiosis are often host-colony specific, with necrotic octocoral tissues exhibiting an enrichment of bacterial taxa such as *Roseobacteraceae* (specifically *Ruegeria*), *Rhodobacteraceae*, *Verrucomicrobiaceae*, and *Flavobacteriaceae* (Keller-Costa et al., 2021; Rubio-Portillo et al., 2021). In addition, environmental stressors have also been associated with alterations in microbial community structure, frequently involving opportunistic or potentially pathogenic genera such as *Vibrio*, *Pseudoalteromonas*, *Ruegeria*, and members of the *Rhodobacteraceae* and *Hyphomonadaceae* families (Prioux et al., 2023; Tignat-Perrier et al., 2022, 2023; Xiang et al., 2022). Notably, in some cases, octocoral microbiomes remained stable despite environmental stressors. In a marine protected area, *Paramuricea clavata* showed no significant shifts in microbiome composition following a heatwave, unlike neighboring species (*Eunicella cavolini*, *Corallium rubrum*), while *Pinnigorgia flava* exhibited resilience under certain DOC enrichments and warming conditions in aquarium experiments (Corinaldesi et al., 2022; Xiang et al., 2022; Zelli et al., 2023). These observations underscore the importance of host-specific factors in shaping microbial assemblages. Indeed, growing evidence supports the concept of phylosymbiosis, whereby microbiome composition correlates with host phylogeny. This pattern is particularly evident within the families *Endozoicomonadaceae* and *Spirochaetaceae*, especially among tropical and temperate octocoral species (Keller-Costa et al., 2021; O’Brien et al., 2020; Pollock et al., 2018; Prioux et al., 2024).

In this context, *Litophyton*, a branching octocoral commonly found in tropical waters, represents a valuable yet underexplored taxon for investigating octocoral-microbe interactions. It inhabits a wide depth range, from shallow habitats (<10 m) to 70 m (Hsu et al., 2020; Liberman et al., 2022; Park et al., 2022; Pupier et al., 2019). This soft coral taxon harbors photosymbionts of the family *Symbiodiniaceae* (Liberman et al., 2022), but it is thought to be predominantly heterotrophic, benefitting from photosynthetic-derived carbon as a nutritional supplement (Hsu et al., 2020). Research on the *Litophyton* microbiome, although still limited, has gained traction recently (Alsharif et al., 2023; Goulet et al., 2008; Hsu et al., 2020; Liberman et al., 2022; Park et al., 2022). While studies by Hsu et al. (2020) and Liberman et al. (2022) focused on *Symbiodiniaceae* communities, Park et al. (2022) and Alsharif et al. (2023) explored the bacterial communities associated with *Litophyton* and the surrounding seawater.

As the field grows, ensuring methodological consistency is critical for robust comparisons across studies and systems. Microbiome profiling is known to be sensitive to methodological variables such as sample preservation and DNA extraction protocols, which can influence microbial diversity estimates and taxonomic representation (Hernandez-Agreda et al., 2018; Weber et al., 2017). However, this issue remains largely unexplored in octocorals. To address this gap, our study investigates how two common sample processing strategies—direct DNA extraction from coral holobiont samples and indirect extraction from microbial pellets—affect the characterization of *Litophyton*-associated prokaryotic communities.

In parallel, there is increasing interest in leveraging ex situ systems to complement field-based studies. More than 200 aquarium exhibition facilities worldwide maintain corals under controlled, in principle unstressed, conditions (The MarineBio Conservation Society, n.d.). The growth and stress responses of octocorals have been studied in small-scale aquaria, primarily within the contexts of the marine aquarium trade or their response to environmental change (Cau et al., 2018; Gómez-Gras et al., 2022; Gugliotti et al., 2019; McCauley et al., 2018, 2020; Previati et al., 2010; Pupier et al., 2019; Simancas-Giraldo et al., 2021; Tignat-Perrier et al., 2022, 2023; Tracy et al., 2020, 2021; Vezzulli et al., 2010; Vollstedt et al., 2020; Wessels et al., 2017). These studies demonstrated that octocorals exhibit species-specific and depth-dependent variations in thermal tolerance, influenced by environmental conditions. Notably, a subset of these studies has specifically addressed octocoral microbiome changes in small-scale aquaria (Bonacolta et al., 2024; McCauley et al., 2018, 2020; Pupier et al., 2019; Rocha et al., 2013; Tignat-Perrier et al., 2022, 2023; Vezzulli et al., 2010; Wessels et al., 2017). However, in contrast with research on hexacorals (e.g., Galand et al., 2018; Pratte et al., 2015), and to the best of our knowledge, no studies have yet compared the microbiomes of octocorals between wild populations and those maintained in large-scale aquarium settings.

The present study aims to explore similarities in the prokaryotic communities associated with *Litophyton* sp. specimens maintained in long-term captivity within an established aquarium ecosystem and those of wild specimens living in natural conditions at a Red Sea reef ecosystem. In parallel, we evaluate how sample processing strategies influence microbial diversity and composition. A key objective of our study was to identify potentially conserved microbial associates across environmental contexts and to determine whether aquaria can serve as repositories for both the host animal and its associated microbiome. These insights contribute to assessing the potential role of aquarium facilities in marine microbiome stewardship, with implications for future strategies aimed at preserving and rehabilitating coral ecosystems under increasing environmental stress.

## 2 Materials and methods

### 2.1 Sampling sites and sample collection

This study investigates the prokaryotic communities associated with *Litophyton* sp. specimens collected from two distinct environments, herein termed “habitats”: (1) a long-term aquarium system at Oceanário de Lisboa, Portugal, and (2) a natural reef environment in the Red Sea, Saudi Arabia.

**Figure 1:**
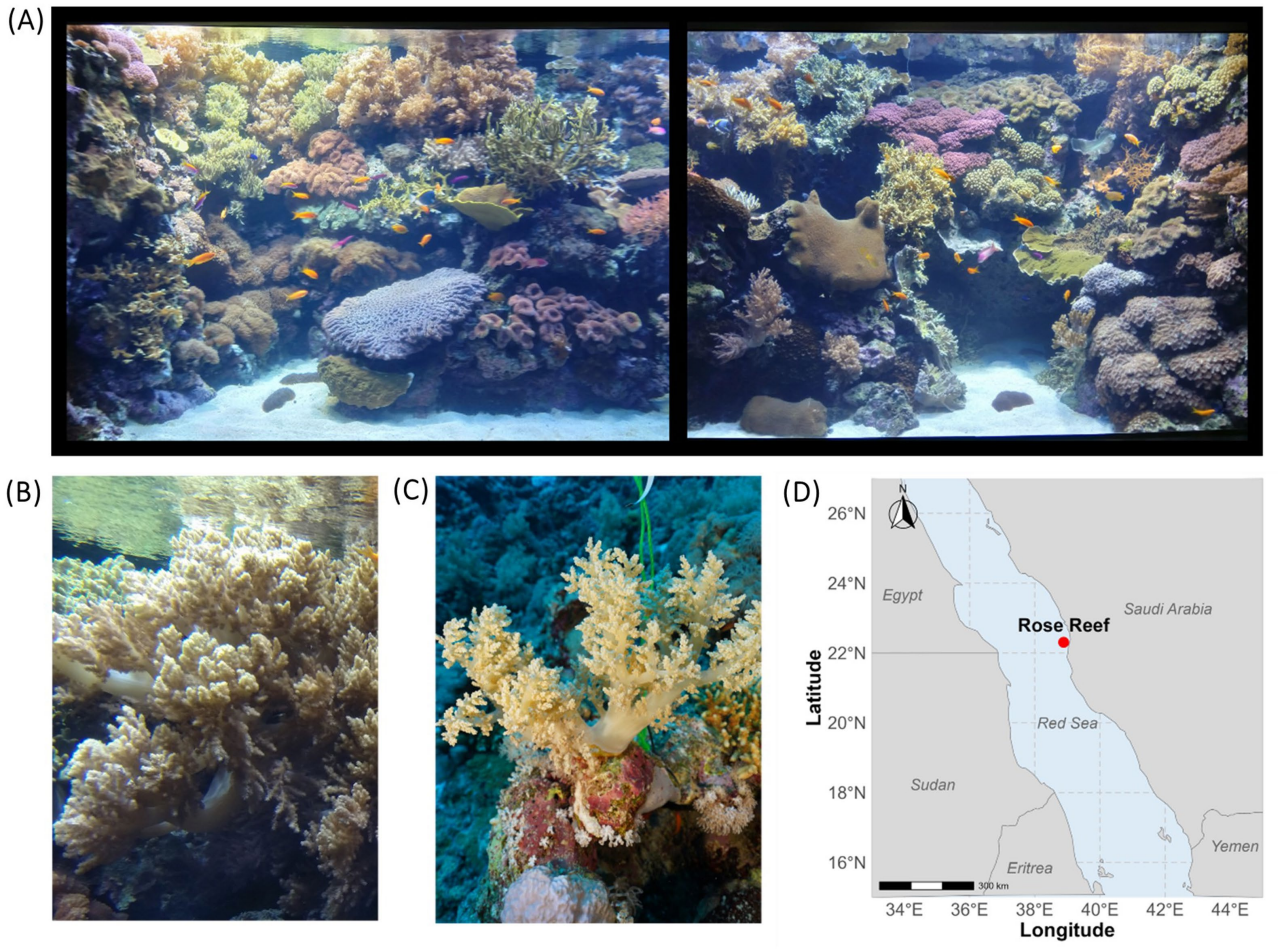
Sampling locations and *Litophyton* specimen overview. **(A)** Tropical coral aquarium at the Oceanário de Lisboa, Portugal where samples were collected for this study. **(B)** Representative *Litophyton* specimen sampled from the coral aquarium at the Oceanário de Lisboa. **(C)** Representative *Litophyton* specimen collected *in situ* from the Red Sea. **(D)** Map indicating the sampling location in the Red Sea, marked with a red dot. Pictures in panels **(A,B)** were taken by Matilde Marques; panel **(C)** by Helena Villela.

The first sampling event took place on April 5th, 2022, in a tropical public aquarium at Oceanário de Lisboa (38°45'49.2''N 9°05'37.0''W), operating since 1998 (Figures 1A,B). The aquarium, with a capacity of 19 m^3^, is equipped with artificial seawater and sediments, and is maintained under stable, controlled conditions with minimal seasonal variation. It operates on a 12:12 light–dark photoperiod and maintains an average water flow of 29 m^3^/h. An additional pump enhances water movement, providing a flow rate of 66-68 m^3^/h. The artificial seawater is kept at 25°C, with pH ranging from 8.15-8.24 and a salinity of 32.9-33.5 ppt. The aquarium ecosystem hosts diverse animals that, alongside multiple tropical hexacoral and octocoral species, include fishes, sea cucumbers, snails, and hermit crabs. The corals, of unknown origin before 1998, are fed every day with live *Artemia nauplii*, enriched with live *Isochrysis* algae, and every other day with coral V powder or *Calanus* copepods. The fish are fed twice a day with a mix of frozen *Mysis* shrimp, frozen brine shrimp (*Artemia*), frozen krill (*Euphasia pacifica*) and every other day with frozen red plankton or frozen cyclops.

Three specimens of *Litophyton* sp. (previously identified as *Nephthea* sp.) were sampled from this aquarium (*N* = 3, OLIT1-3). Coral fragments (min. 3 cm) were cut with a sterile diving knife and immediately placed into individual Ziploc plastic bags containing surrounding aquarium seawater. In addition, triplicate samples of surrounding seawater (*N* = 3, OASW1-3) and sediment (*N* = 3, OSED1-3) were collected in sterile containers. All samples were kept in a cooling box and transported to the laboratory within 1 h, where they were immediately processed for total community DNA (TC-DNA) extraction.

The second sampling event took place on October 24th, 2022, by SCUBA diving at Rose Reef, located in the central Red Sea (22°18'22.8''N 38°53'07.2''E; Figures 1C,D), under a sampling permit for coral collection for research purposes (IBEC protocol number 22ibec003). Rose Reef is a near-vertical wall reef that extends over 30 m in depth and supports a diverse assemblage of hexa- and octocorals, alongside a rich fish community, and occasional sightings of sharks. At the time of sampling, the seawater temperature was 31°C, with a pH of 7.89. While the Red Sea is known to exhibit seasonal changes in environmental parameters, the objective of this study was to compare natural octocorals with those maintained in an aquarium under stable conditions, rather than to investigate seasonal dynamics. Therefore, sampling was conducted in October due to logistical constraints.

Three *Litophyton* sp. specimens were collected by SCUBA diving (*N* = 3, RLIT1-3), from coral colonies located along the reef wall at 20- and 24-m depth (Supplementary Table S1). Sampling was restricted to macroscopically healthy colonies, showing no visible signs of tissue degradation, disease, bleaching, or loss of turgidity. Coral fragments (min. 3 cm) were cut off from the colonies with sterile scissors and individually placed in Ziploc plastic bags containing surrounding seawater, immediately upon collection. Triplicate samples of surrounding seawater (*N* = 3, RSW1-3) were taken at approximately 3 m from coral colonies, and surface sediment (*N* = 3, RSED1-3) was collected from the seafloor directly adjacent to or within 1 m distance from the coral colonies. All samples were collected in sterile containers, stored in a shaded cooling box, and transported to the laboratory within 4 h, where they were immediately processed for TC-DNA extraction.

Detailed procedures regarding octocoral molecular identification and phylogenetic inference are provided as Detailed Methodology in Supplementary Material File 2.

### 2.2 Sample processing, DNA extraction and sequencing

Sample processing was performed under sterile conditions, in a biological safety flow cabinet, as earlier described (Keller-Costa et al., 2017, 2021). Up to 4 g of each coral sample, comprising mucus, tissue and sclerites (as this species lacks a calcium carbonate skeleton), was collectively processed to capture the overall holobiont-associated microbial community, since compartmentalized analyses were beyond the scope of this study. Coral fragments were rinsed with sterile Calcium and Magnesium Free Artificial Seawater (CMFASW: 27 g L^−1^ NaCl, 1 g L^−1^ Na_2_SO_4_, 0.8 g L^−1^ KCl and 0.18 g L^−1^ NaHCO_3_) to clean fragments, and eventual epibionts were removed. Half a gram (0.5 g) of each sample, as defined above, was aseptically cut and immediately stored at −80°C until TC-DNA extraction (aquarium: OLIT1-3; Red Sea: RLIT1-3) to preserve the integrity of the holobiont-associated microbial community. One gram (1.0 g) of each sample was further homogenized in 9 ml sterile CMFASW using a sterile mortar and pestle. The resulting homogenate was vortexed at maximum speed for 1 min with sterile 2 mm glass beads (Supelco), followed by 2 min of centrifugation at 500 *g* at 4°C. The resulting supernatants were transferred into new 15 mL tubes and centrifuged for 30 min at 10,000 *g* at 4°C. The supernatant was discarded, and the resulting coral-derived microbial pellets (aquarium: MPOLIT1-3; Red Sea: MPRLIT1-3) were stored at -80°C until TC-DNA extraction. For each seawater sample, c.a. 1 L was filtered through a sterile 0.22 μm nitrocellulose membrane filter (MF-Millipore; 47 mm) using a vacuum pump at c.a. 15 cm Hg. The filters were then aseptically cut in half, and each filter piece was stored in sterile 2 ml Eppendorf tubes at −80°C until TC-DNA extraction. Per sediment sample, c.a. 0.5 g was weighed and stored in sterile 2 ml Eppendorf tubes at −80°C until TC-DNA extraction.

TC-DNA extraction was performed for all samples using the DNeasy PowerSoil Pro Kit (Qiagen), following the manufacturer’s protocol. Two negative control samples were processed alongside the biological samples. The first was an extraction kit control (i.e., a control using the extraction kit without any biological material, also known as the “kitome”), and the second was a filter control (i.e., an extraction performed on a sterile 0.22 μm nitrocellulose membrane filter). DNA concentration was measured using an Invitrogen Qubit 4 (aquarium samples) and 3 (Red Sea samples) Fluorometer (Fisher Scientific) and the Qubit dsDNA BR (Broad-Range) and HS (High Sensitivity) Assay kits (Supplementary Table S1). DNA was stored at −20°C until being sent for amplicon library preparation and sequencing using Illumina’s standard “16S Metagenomic Sequencing Library Preparation” protocol (Illumina, 2013), MiSeq Reagent Kit v3 and following a 2 × 300 bp (600 cycles) paired-end approach, on an Illumina MiSeq platform at StabVida (Lisbon, Portugal). The hypervariable V4 region of the 16S rRNA gene found in bacteria and archaea was targeted for PCR amplification using the modified primers 515F (5'-GTGYCAGCMGCCGCGGTAA-3') and 806R (5'-GGACTACNVGGGTWTCTAAT-3') (Apprill et al., 2015; Parada et al., 2016). This primer set is recommended by the Earth Microbiome Project^1^ and corrects for amplification bias of important marine groups such as clade SAR11 and *Thaumarchaeota*. MiSeq sequencing produced a total of 7,272,526 16S rRNA gene reads ranging from 12,906 to 648,684 reads per sample (Supplementary Table S2). The fastq files containing the raw sequencing data have been deposited in NCBI’s Sequence Read Archive (SRA), under the BioProject accession number PRJNA1256069.

### 2.3 Bioinformatics processing of 16S rRNA gene amplicon data

The raw reads from 16S rRNA gene amplicon sequencing were processed into ASVs in R, using the DADA2 (Divisive Amplicon Denoising Algorithm) R package v1.34.0 (Callahan et al., 2016). Primer sequences were removed by trimming at the 5' -end of each read (19 bp and 20 bp on the forward and reverse reads, respectively). Forward and reverse reads were truncated to 230 nt (forward) and 180 nt (reverse) and filtered using default parameters, except for the maxEE and truncQ parameters, which were set to 5. Error rates were computed to identify unique sequences, forward and reverse reads were merged, and chimeras were filtered out of the final ASV table. Chimeras accounted for about 2.73% of all reads. Taxonomy was tentatively assigned to a total of 17,853 ASVs, using the naive Bayes algorithm (Wang et al., 2007) with the SILVA reference database v138.2 (Quast et al., 2013; Yilmaz et al., 2014). Taxonomic assignments were subsequently manually curated, where necessary, to comply with the International Code of Nomenclature of Prokaryotes (ICNP) and the List of Prokaryotic names with Standing in Nomenclature (LPSN) (Parte et al., 2020). ASVs with sequences shorter than 200 bp or longer than 270 bp were filtered out (leading to the removal of 757 ASVs). Moreover, ASVs not assigned to prokaryotes at domain level (356 Not Assigned -NA- and 869 Eukaryota ASVs), and those assigned to chloroplasts (191 ASVs) and mitochondria (403 ASVs) were removed. The two negative control samples included 52 ASVs from the “filter control” and 10 ASVs from the “kitome” and served to detect contaminant ASVs in the biological samples. Negative control ASVs that were found in any of the biological samples were proportionally subtracted from the corresponding sample abundances. ASVs detected in the “filter control” were proportionally subtracted from all seawater samples, while those identified in the “kitome” were proportionally subtracted from all biological samples. Singletons (5 reads pertaining to 5 ASVs) and doubletons (1,282 reads corresponding to 641 ASVs) were excluded from subsequent analyses to ensure data robustness. Due to the high proportion of ASVs without taxonomic assignments across multiple levels, these ASVs (from phylum to genus) were subjected to BLAST analysis on NCBI. This process identified 16 ASVs as mitochondrial DNA, which were subsequently removed (372,590 reads). After completing these filtration and curation steps, a total of 1,212,651 reads were retained, corresponding to 14,689 unique ASVs across 24 samples (Supplementary Table S3). Supplementary Table S4 provides a detailed breakdown of the number of reads and ASVs per sample that passed through the different steps of the pipeline and filtration processes.

### 2.4 Microbiome composition, diversity and statistical analysis

Taxonomic composition, and alpha- and beta-diversity analyses, were conducted using the R phyloseq, ggplot2, vegan, tidyverse, stats, agricolae and pairwiseAdonis packages (de Mendiburu, 2023; Martinez Arbizu, 2017; McMurdie and Holmes, 2013; Oksanen et al., 2024; R Core Team, 2023; Wickham, 2016; Wickham et al., 2019). To account for differences in sequencing depth across samples, rarefaction was applied to standardize the sequencing depth to 466 reads per sample when including sample RLIT1, and to 2,548 reads per sample when excluding RLIT1, for alpha-diversity estimates. Alpha-diversity was assessed using the species richness measures (ASV counts) and the Shannon-Wiener diversity index. To test for significant differences in species richness, normality and equal variance were first assessed with the Shapiro–Wilk and Levene’s tests, respectively. Since data was normally distributed with equal variance, a two-way ANOVA was performed with habitat and biotope as fixed factors, including their interaction, followed by Tukey’s Honest Significant Difference (HSD) post-hoc test.

Beta-diversity was visualized using Non-Metric Multidimensional Scaling (NMDS) applied to Bray–Curtis dissimilarity measures calculated from non-rarefied, Hellinger-transformed ASV abundance data. The Hellinger transformation consists of taking the square root of relative abundances, being suitable for the preparation of compositional data for multivariate statistics as it performs well with sparse data and varying sampling depths (Legendre and Gallagher, 2001). To identify the ASVs contributing most to differences in community composition among sample groups, a Similarity Percentage (SIMPER) analysis was performed. The 10 ASVs with the highest contributions to group dissimilarities were visualized as vectors on the NMDS ordination plot to illustrate associations between biotopes (*Litophyton*, seawater, sediment), habitats (Red Sea versus Aquarium), and symbiont taxa. Furthermore, unrestricted full-factorial and pairwise permutational multivariate analysis of variance (PERMANOVA) were used to evaluate the influence of habitat and biotope on prokaryotic community structure, using 9,999 permutations and correcting for multiple comparisons with the Bonferroni method.

To assess differences in individual ASV abundance, the Anderson-Darling test was first used to assess the normality of non-rarefied ASV relative abundance profiles, which were determined to be non-normal (*p*-value < 0.05). Accordingly, differential abundance analyses were conducted to identify ASVs that differed significantly in relative abundance: (i) between biotopes within the same habitat, (ii) across biotopes regardless of habitat, and (iii) within the same biotope across different habitats. These comparisons were performed using the non-parametric Kruskal-Wallis test, and a two-sided Welch’s t-test, implemented in STAMP v2.1.3 (Parks et al., 2014). For both tests, significance was assessed using raw *p*-values (*p*-value < 0.05), as applying multiple testing corrections (e.g., Benjamini-Hochberg) resulted in no ASVs meeting the significance threshold. To mitigate the risk of false positives and highlight biologically meaningful differences, an additional effect size filter was applied by setting the “ratio of proportions” threshold to 2.0.

The core prokaryotic microbiome of each biotope was defined as those ASVs occurring in all samples (non-rarefied) of a given biotope. Additionally, to capture taxa that are highly prevalent but not strictly ubiquitous, ASVs present in at least 65% of seawater samples and 80% of octocoral samples were also identified. ASVs meeting this second threshold and shared between both habitats were defined as the “expanded core microbiome” of Litophyton and seawater samples. This relaxed threshold provides a balance between ecological relevance and statistical robustness, allowing the inclusion of microbes that occur at high, but not universal, prevalence due to natural individual, spatial or temporal variability. Similar relaxed thresholds have been applied in other microbiome studies (Aguirre de Cárcer, 2018; Clever et al., 2022) to generate a more inclusive yet robust representation of the stable microbial community while still filtering out low-frequency taxa.

To assess the effect of the sample processing strategy on octocoral microbial diversity, both alpha- and beta-diversity metrics were computed and compared considering only *Litophyton* samples. For alpha-diversity, raw ASV counts were rarefied to 2,548 reads per sample to standardize sequencing depth. Given that one interaction group, direct DNA extraction from Red Sea samples, included only two replicates, we could not reliably assess normality when testing interaction effects involving habitat. For group comparisons, we first assessed assumptions of normality and homogeneity of variance using Shapiro–Wilk and Levene’s tests, respectively. When both assumptions were met (*p*-value > 0.05), ANOVA was applied. When assumptions were violated (*p*-value < 0.05) or could not be reliably assessed due to small group sizes, the non-parametric Kruskal-Wallis test was used. For beta-diversity, community composition was analyzed using Hellinger-transformed ASV counts as described above. An unrestricted full-factorial PERMANOVA, with 9,999 permutations, was used to test the influence of sample processing strategy, and its interaction with habitat on prokaryotic community structure. Because PERMANOVA assumes homogeneous dispersion, multivariate dispersion was examined using PERMDISP. To quantify how much of the total variation in community structure was explained by sample processing strategy, we applied Canonical Analysis of Principal Coordinates (CAP), with significance assessed by permutation ANOVA. Finally, we repeated the PERMANOVA while accounting for any dispersion differences to confirm that the sample processing strategy effect was not driven solely by unequal dispersion.

### 2.5 16S rRNA gene-based phylogenetic analyses of *Endozoicomonas* sequences

A phylogenetic tree was constructed using all the 12 ASVs assigned to the *Endozoicomonas* genus from this study, nearly full length 16S rRNA gene sequences of six *Endozoicomonas lisbonensis* isolates cultured from two of the here studied aquarium *Litophyton* (OLIT1, OLIT2) samples (Marques et al., 2025; Da Silva et al., 2025), and all validly published *Endozoicomonas* type strains. Sequences from three *Kistimonas* type strains, the closest genus to *Endozoicomonas*, were used as an outgroup to root the tree. Alignment was performed with the CLUSTALW algorithm, and the optimal evolutionary model was identified in MEGA version X (Kumar et al., 2018) using the “find best DNA/Protein Model” function. The Kimura 2-parameter model (Kimura, 1980) with a discrete Gamma distribution (eight categories, +G parameter = 0.3461, [+I], 55.84%) was selected. A Maximum-Likelihood tree was then determined with 1,000 bootstrap replicates. The tree with the highest log likelihood (−822.80) was used with a total of 253 nucleotide positions in the final dataset. Using the “partial deletion” option, positions with less than 85% site coverage (allowing < 15% gaps, missing data, or ambiguous bases) were eliminated. The calculated tree was visualized and styled using iTOL (Interactive Tree Of Life) v7.1 (Letunic and Bork, 2024) and Inkscape (Inkscape, 2020).

## 3 Results

### 3.1 Dataset overview

The final dataset analyzed in this study comprised 24 samples, including 12 *Litophyton*, six sediments, and six seawater samples. Post-filtering, these samples collectively yielded a total of 1,212,651 prokaryotic 16S rRNA gene reads, with a mean of 50,527 reads per sample and a standard deviation of 44,755. Rarefaction curves plateaued for all samples (Supplementary Figure S1), indicating sufficient sequencing depth. However, sample RLIT1 presented a comparatively lower read count at 466 reads. To ensure the robustness of our findings, we performed the main analyses with and without this sample. Results presented in the main text exclude sample RLIT1, while analyses including it are available in the Supplementary materials and are briefly addressed in the Results section.

The 11 *Litophyton* samples considered here (five from the Red Sea, six from the aquarium) accounted for 102,725 reads (mean ± SD: 9,339 ± 5,448 reads; Supplementary Table S4), with individual sample counts ranging from 2,548 reads in OLIT3 to 20,966 reads in MPRLIT3. In the *Litophyton* samples, 287 ASVs were identified, of which 128 ASVs were classified into 80 different genera, while the remaining ASVs could not be classified at genus level. Sediment samples accounted for a total of 528,093 reads (mean ± SD: 88,016 ± 11,430 reads; Supplementary Table S4), with counts ranging from 75,392 in OSED3 to 109,899 reads in RSED2. These samples encompassed 10,416 ASVs, of which 3,097 were classified into 533 genera. Seawater samples accounted for 581,367 reads (mean ± SD: 96,895 ± 27,272 reads; Supplementary Table S4), with read counts ranging from 56,523 in OASW2 to 141,088 reads in OASW3. These seawater samples contained 4,848 ASVs, with 2,896 ASVs classified into 523 genera.

The taxonomic affiliation of the octocorals sampled was validated through the analysis of the mitochondrial Mutator S (mtMutS; formerly msh1) and Cytochrome Oxidase Subunit I (COI) gene sequences, which were compared against voucher sequences from the *Litophyton* genus and the tropical octocoral genus *Sclerophytum* as outgroup. Both COI (Supplementary Figure S2) and mtMutS (Supplementary Figure S3) analyses confirmed that the sampled octocorals belong to the *Litophyton* genus.

### 3.2 Prokaryote richness and diversity patterns are maintained in aquarium facilities

**Figure 2:**
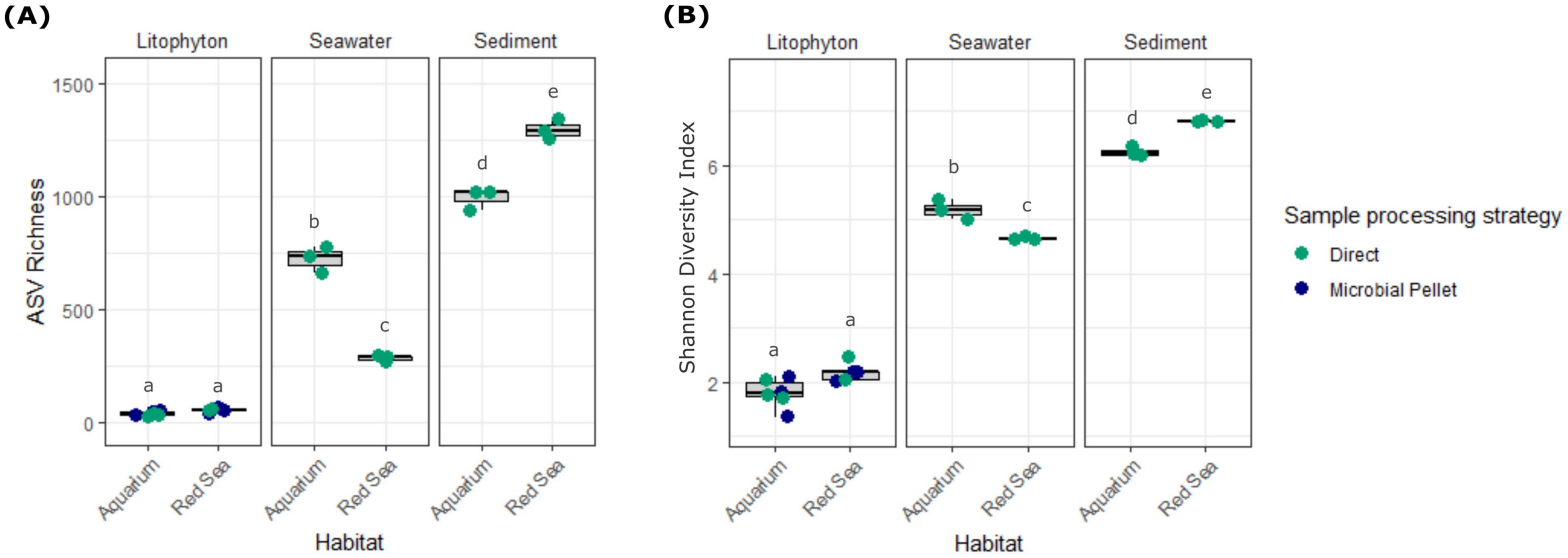
Alpha-diversity analysis of prokaryotic communities associated with *Litophyton* sp. and the surrounding environment. **(A)** ASV richness and **(B)** Shannon-Wiener diversity index are compared across biotopes using a rarefied dataset with a threshold of 2,548 sequences. The coloring of the dots indicates the sample processing strategy: green—direct DNA extraction from holobiont sample, blue—DNA extracted from microbial pellets. Different letters above box plots indicate significant differences between biotope-habitat groups per index (ANOVA followed by a Tukey’s HSD post-hoc test; *p*-value < 0.05). For alpha diversity analyses performed with a rarefaction threshold of 466 sequences per sample, including sample RLIT1, please consult Supplementary Figure S4.

In both the artificial aquarium habitat and the natural Red Sea environment, prokaryote ASV richness was biotope-specific, exhibiting consistent trends across habitats (Figure 2A). Considering the rarefied data (threshold at 2,548 reads per sample), *Litophyton* consistently showed the lowest richness in both habitats, with an observed richness of 39 ± 7 (mean ± SD) ASVs for the aquarium and of 56 ± 10 (mean ± SD) for the Red Sea (Supplementary Table S5). In contrast, seawater samples exhibited approximately a 5- to 19-fold increase in richness, reaching 724 ± 51 (mean ± SD) ASVs for the aquarium and 284 ± 12 (mean ± SD) ASVs for the Red Sea (Supplementary Table S5). Sediments showed the highest richness, with 992 ± 35 (mean ± SD) ASVs for the aquarium and of 1,299 ± 33 (mean ± SD) for the Red Sea (Supplementary Table S5), corresponding to a 23- to 25-fold increase relative to *Litophyton*. A two-way ANOVA revealed a significant effect (*p*-value < 0.05) of biotope, habitat, and of the biotope-habitat interaction on ASV richness (Supplementary Table S6). Tukey’s HSD post-hoc test showed that all pairwise comparisons were statistically significant (*p*-value < 0.05), except for *Litophyton* from the aquarium versus the Red Sea (*p*-value > 0.05; Supplementary Table S6). The 95% confidence intervals for significant comparisons did not include zero, confirming the robustness of these differences. A similar trend was observed for the Shannon-Wiener diversity index (Figure 2B), which increased progressively from *Litophyton*, to seawater, and then sediments. Patterns of variation mirrored those observed for ASV richness (Supplementary Table S6). When sample RLIT1 was included in the analysis, and a rarefaction threshold of 466 reads per sample was applied, overall alpha-diversity trends remained the same (Supplementary Figure S4; Supplementary Table S6).

### 3.3 Sample processing influences prokaryotic community composition of *Litophyton*

Prokaryotic alpha-diversity was assessed in *Litophyton* samples across two habitats (aquarium vs. Red Sea) and two sample processing strategies: direct DNA extraction from holobiont samples vs. indirect DNA extraction from microbial pellets prepared from holobiont samples. For alpha-diversity, both ASV richness and Shannon diversity indices were computed, and no significant differences were observed in either metric with respect to sample processing strategy or the interaction between sample processing strategy and habitat (*p*-value > 0.05 in all cases; Supplementary Table S7). In contrast, analysis of beta-diversity using Bray-Curtis dissimilarities on Hellinger-transformed data revealed a significant effect of sample processing strategy on prokaryotic community composition, as indicated by an unrestricted PERMANOVA (*p*-value = 0.0003, *F* = 1.4885, R^2^ = 0.0696), while the habitat-processing strategy interaction had no significant effect (*p*-values > 0.05; Supplementary Table S7). Although NMDS did not clearly separate the groups by sample processing strategy (data not shown), an assessment of beta dispersion revealed noticeable differences in within-group variability. Specifically, samples from the direct DNA extraction group exhibited greater dispersion (Supplementary Table S7), with a higher average distance to the group median (0.3587), compared to the microbial pellets group (0.2564). This observation was supported by a test for homogeneity of group dispersions (PERMDISP), which identified significant differences in multivariate dispersion between sample processing strategies (*p*-value = 0.046), indicating heterogeneity in community variability across groups (Supplementary Table S7). To further elucidate these patterns, CAP was performed, revealing that the sample processing strategy accounted for approximately 56% of the total variation in community structure (Supplementary Table S7). The CAP model was statistically supported by permutation ANOVA (*p*-value = 0.004, *F* = 11.29; Supplementary Table S7). Moreover, a PERMANOVA adjusted for differences in dispersion confirmed that the observed effect of sample processing strategy was marginally non-significant (*p*-value = 0.0527, *F* = 5.50, R^2^ = 0.38; Supplementary Table S7).

### 3.4 *Litophyton* and sediment samples maintain dominant bacterial taxa in aquarium settings

**Figure 3:**
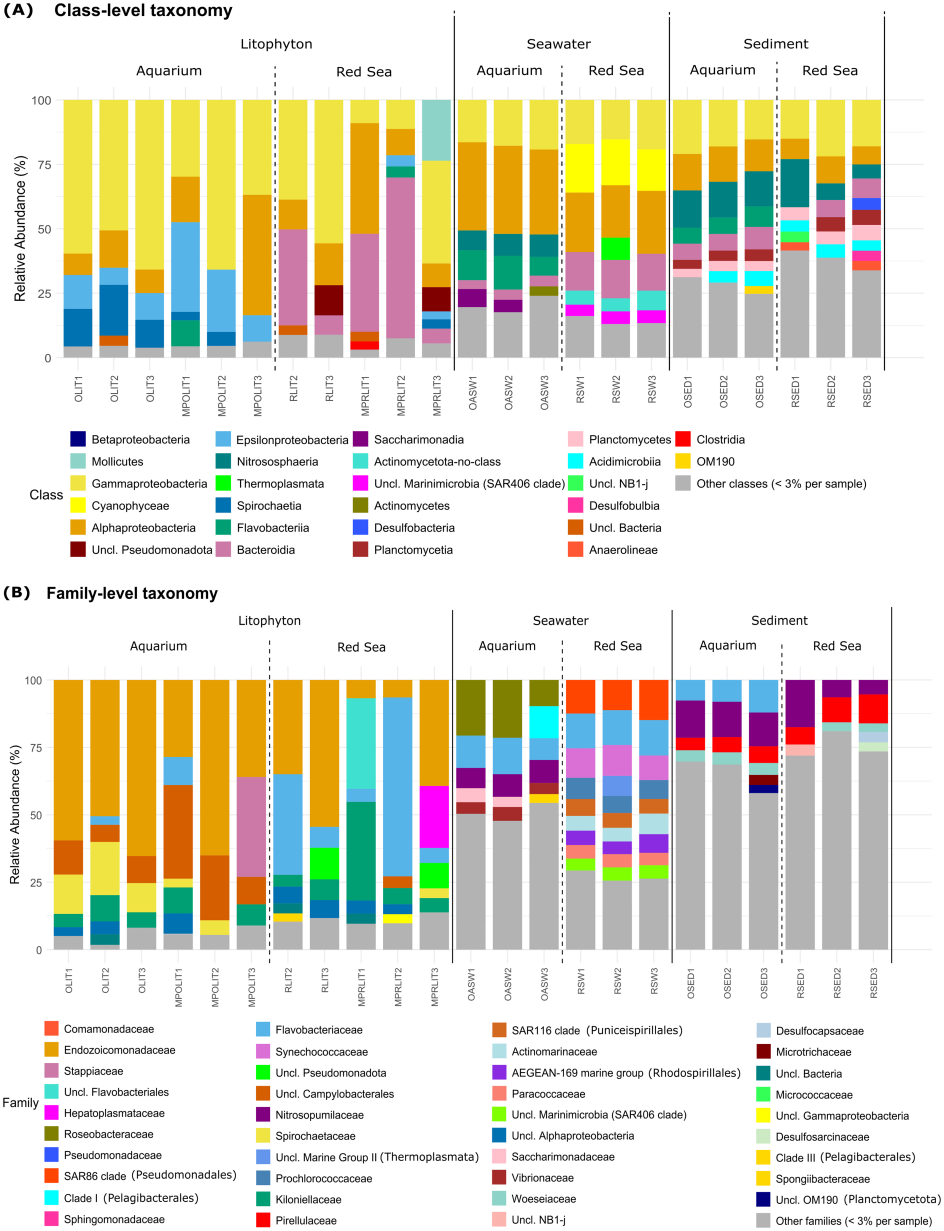
Prokaryotic community composition of *Litophyton*, seawater and sediment samples. **(A)** class-level and **(B)** family-level prokaryotic community profiles are shown for samples from the aquarium: *Litophyton* (direct DNA extraction: OLIT1–OLIT3; microbial pellets: MPOLIT1-MPOLIT3), artificial seawater (OASW1–OASW3), and sediments (OSED1–OSED3), as well as from the Red Sea: *Litophyton* (direct extraction: RLIT2–RLIT3; microbial pellets: MPRLIT1-MPRLIT3), seawater (RSW1–RSW3), and sediments (RSED1–RSED3). Relative abundances are presented for taxa contributing more than 3% of the total reads per sample. Taxa with lower relative abundances were grouped under “Other classes” or “Other families”.

The *Gammaproteobacteria* class typically dominated the prokaryotic communities of *Litophyton* specimens both in the aquarium (average relative abundance of 51%) and the Red Sea (31%), although the Red Sea presented higher variability between samples (Figure 3A). *Alphaproteobacteria* were also identified in all *Litophyton* samples, although with a relative abundance below 3% in MPOLIT2. *Epsilonproteobacteria* exceeded 3% relative abundance in all aquarium samples, while in Red Sea samples, except MPRLIT1—where they were absent—remained relatively close to this threshold. *Spirochaetia* were similarly present in all aquarium samples, although MPOLIT3 showed a relative abundance below 3%. In the Red Sea, *Spirochaetia* consistently remained below the 3% threshold in all samples, apart from MPRLIT3. *Bacteroidia* were present in all *Litophyton* samples, although their relative abundance remained below 3% in all aquarium samples.

All seawater samples were dominated by *Alphaproteobacteria* (average relative abundance of 34% in the aquarium and 23% in the Red Sea), along with *Gammaproteobacteria* (average relative abundance of 18% in the aquarium and 17% in the Red Sea). *Flavobacteriia*, *Cyanophyceae*, and *Nitrososphaeria* were present in all seawater samples, although *Flavobacteriia* and *Nitrososphaeria* exceeded 3% only in aquarium seawater samples, while *Cyanophyceae* exceeded 3% only in Red Sea seawater samples. The class *Thermoplasmata* was present in five out of six seawater samples (not present in OASW3), always remaining below 3% in aquarium samples. Unique to aquarium seawater samples was *Saccharimonadia* (formerly TM7), which exceeded 3% relative abundance in two out of three aquarium samples but was absent from the Red Sea. Conversely, unclassified *Marinimicrobia* (SAR406 clade) was unique to Red Sea seawater samples, exceeding 3% relative abundance in all samples and being absent from the aquarium.

Sediment samples exhibited the highest diversity of bacterial classes with relative abundances below 3%. Common to all sediment samples across habitats and typically above 3% relative abundance were *Gammaproteobacteria*, *Bacteroidia*, *Alphaproteobacteria*, *Plantomycetes*, *Nitrososphaeria* and *Acidimicrobiia*. The uncultivated class OM190 (*Planctomycetota*) appeared in all sediment samples but exceeded 3% relative abundance in only one aquarium sample (OSED3). Similarly, the class *Anaerolineae* (*Choloroflexota*) was detected in all sediment samples but surpassed 3% in only two Red Sea samples (RSED1 and RSED3).

### 3.5 *Endozoicomonadaceae* bacteria dominate aquarium-kept *Litophyton*

A closer look at the family-level taxonomic profiles (Figure 3B) revealed that taxonomic composition was more variable among Red Sea than aquarium *Litophyton* samples. The *Endozoicomonadaceae* family was present in all *Litophyton* samples. This family dominated the aquarium *Litophyton* samples, reaching up to 65% in samples OLIT3 and MPOLIT2. Common to all aquarium *Litophyton* samples, typically with a relative abundance above 3% per sample, were also the *Spirochaetaceae* and *Kiloniellaceae* families, as well as several ASVs classified up to the order *Campylobacterales,* and to the class *Alphaproteobacteria*, which were also observed in Red Sea *Litophyton* samples. Surprisingly, the *Stappiaceae* family dominated sample MPOLIT3 but presented a relative abundance below 3% in the remaining microbial pellet-derived aquarium *Litophyton* samples, and was entirely absent in *Litophyton* aquarium samples subjected to direct DNA extraction. The dominant families of the *Litophyton* samples from the Red Sea varied between specimens and sample processing strategies. Specimen 2 was dominated by the family *Flavobacteriaceae*, which displayed 37% relative abundance using the direct DNA extraction procedure (RLIT2), with a marked increase to 66% when the microbial pellet extraction (MPRLIT2) procedure was used. *Endozoicomonadaceae*, present at 35% relative abundance in RLIT2, was the second most abundant family in this sample. In specimen 3, *Endozoicomonadaceae* was most dominant, representing 54% in the direct DNA extraction (RLIT3) and 39% in the microbial pellet (MPRLIT3). The *Kiloniellaceae* family, which was detected in all aquarium *Litophyton* samples, was also present in all Red Sea *Litophyton* samples with a relative abundance above 3% and dominating MPRLIT1 (37%). The *Spirochaetaceae* family was similarly found in all Red Sea samples, as well as in aquarium samples, though its overall abundance remained below 3%.

Seawater samples showed greater variability between habitats at family level, with aquarium artificial seawater being dominated by taxa of low (< 3%) relative abundance (Figure 3B). The family *Roseobacteraceae* was among the dominant families in aquarium seawater, with an average relative abundance of 17%, followed by *Flavobacteriaceae* (11%), *Nitrosopumilaceae* (8%), *Vibrionaceae* (5%), Clade I (*Pelagibacterales*; 4%) and *Saccharimonadaceae* (3%). Contrastingly, Red Sea seawater was dominated by *Synechococcaceae* (10%), *Flavobacteriaceae* (13%) and the SAR86 clade (13%), the latter being unique to Red Sea samples. *Nitrosopumilaceae* and *Vibrionaceae* were also present in all Red Sea seawater samples, though both had an average abundance below 1%. Additional families unique to Red Sea seawater, with a relative abundance above 3% per sample, included the AEGEAN-169 marine group, SAR116 clade, *Actinomarinaceae* and unclassified *Marinimicrobia* (SAR406 clade).

Sediment samples from both habitats were dominated by a majority of families with low (< 3%) relative abundances (Figure 3B). In aquarium sediment samples, *Nitrosopumilaceae* was the most abundant family (13%), followed by *Flavobacteriaceae* (9%), *Pirellulaceae* (5%) and *Woeseiaceae* (4%). *Pirellulaceae* (9%) and *Nitrosopumilaceae* (10%) were the most abundant families across all Red Sea sediment samples.

**Figure 4:**
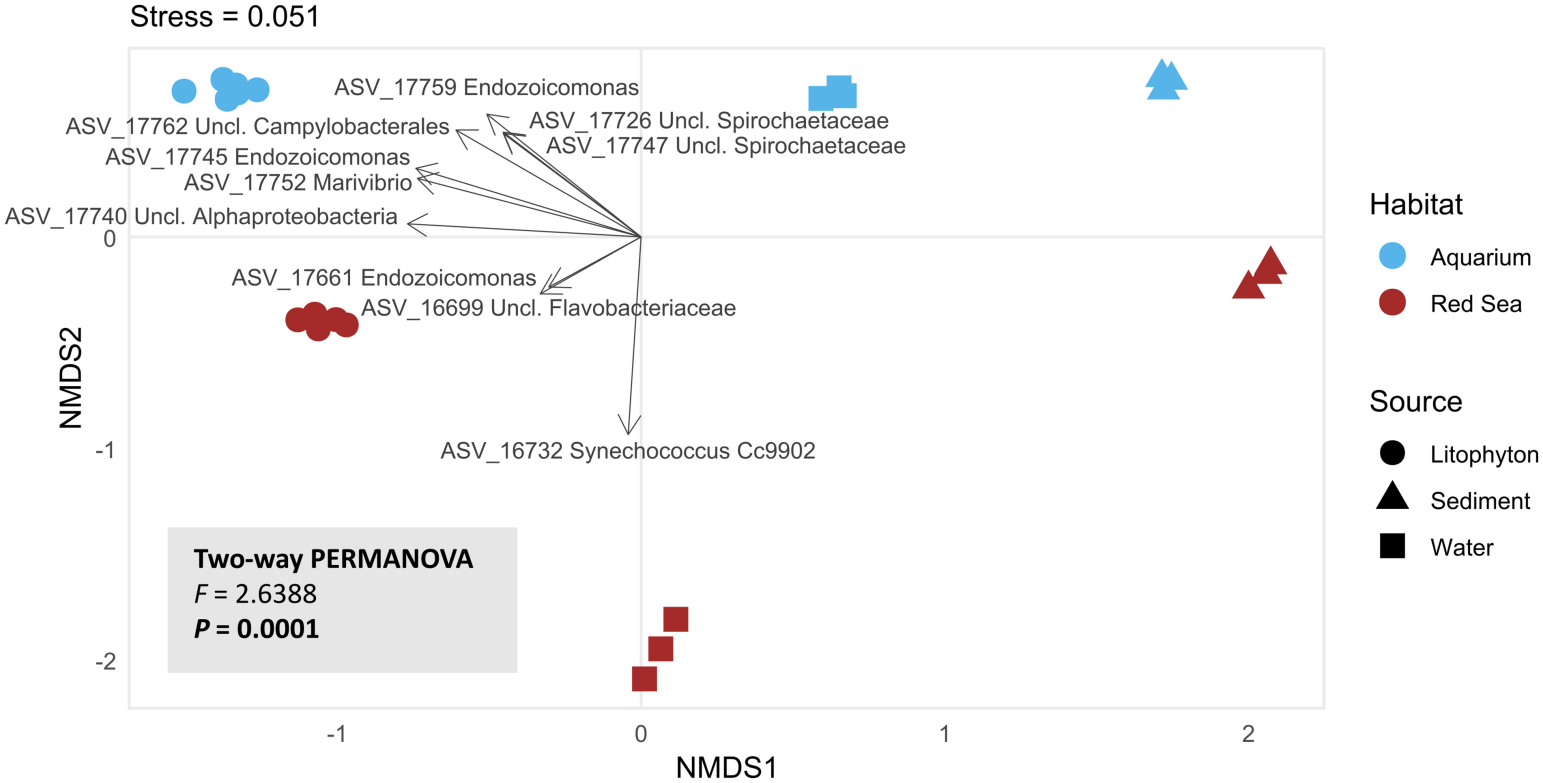
Multivariate analysis of the prokaryotic community profiles. Non-Metric Multidimensional Scaling (NMDS) ordination plot at the ASV level. The plot shows the ordination based on Bray–Curtis similarity matrix calculated from (non-rarefied) Hellinger-transformed abundance data. Aquarium and Red Sea samples are represented by blue and red symbols, respectively. Biotopes are indicated by different shapes: circles for *Litophyton* (RLIT2-3, MPRLIT1-3, OLIT1-3, MPOLIT1-3), triangles for sediments (OSED1-3, RSED1-3), and squares for seawater (OASW1-3, RSW1-3). Black arrows represent the top 10 ASVs contributing most to prokaryotic community dissimilarities, as identified by SIMPER analysis. Differences in prokaryotic community composition were assessed using an unrestricted full-factorial PERMANOVA with 9,999 permutations to evaluate the effects of habitat, biotope, and their interaction, followed by pairwise PERMANOVA analyses to investigate specific group-level differences. An NMDS plot including sample RLIT1 is provided in the Supplementary Figure S5.

In agreement with taxonomic profiling (Figure 3), ordination analysis performed at the ASV level revealed distinct prokaryotic community structures between biotopes (Figure 4; Supplementary Figure S5). Although differences in community composition were detected between DNA extracted directly from the holobiont and from microbial pellets, these variations were relatively minor compared to the pronounced differences observed between *Litophyton* samples and those from seawater or sediments. Therefore, the two sample processing strategies were grouped together in Figure 4 to represent the *Litophyton*-associated prokaryotic community as a single category. Despite a clear difference between habitats within the same biotope, the prokaryotic communities of *Litophyton* and sediments exhibited greater similarity across habitats than those of seawater, as visualized using NMDS (Figure 4). PERMANOVA revealed that prokaryotic communities in seawater and sediment differed more across habitats than those in *Litophyton,* as indicated by higher *F*-values and R^2^ estimates in seawater and sediment comparisons (Supplementary Table S6). The strongest difference was observed between Red Sea seawater and aquarium artificial seawater, which had the highest *F*-value (3.17) and *R^2^* (0.44), indicating notable divergence in microbial composition between them. Notably, aquarium seawater samples clustered closer to aquarium sediment samples than to Red Sea seawater samples. For both ordination settings tested (Figures 4; Supplementary Figure S5), the stress values were well below 0.1, indicating that the reduced-dimensional representation accurately captured the underlying community structure.

### 3.6 Distinct ASV abundance distributions across biotopes and habitats underpin biotope-specific assembly of prokaryotic communities

Differential analysis of ASVs between biotopes within the aquarium habitat revealed distinct taxa associated with each biotope (Supplementary Figure S6). Pairwise comparisons using Welch’s t-test revealed 15 ASVs possessing significantly different abundance distributions between *Litophyton* and sediments, 21 between *Litophyton* and artificial seawater, and 149 between artificial seawater and sediments (Supplementary Figure S6). Remarkably, *Endozoicomonas* ASVs 17759 and 17745 were significantly enriched in *Litophyton* compared to both sediment and artificial seawater (Figure 4; Supplementary Figures S6A,B). Additionally, *Litophyton* was characterized by a higher abundance of *Marivibrio* (ASV 17752), unclassified *Campylobacterales* (ASV 17762) and unclassified *Alphaproteobacteria* (ASVs 17740 and 17741) ASVs in comparison with seawater and sediments. Except for ASV 17741, these five ASVs were among the top 10 contributors to community dissimilarity across all sample groups, as identified by SIMPER analysis (Figure 4; Supplementary Figure S5). In contrast, aquarium sediments were distinguished by the genera *Seonamhaeicola* (ASV 17793), *Woeseia* (ASV 15844), *Ruegeria* (ASV 17384) and *Cenarchaeum* (ASV 16125) and unclassified *Nitrosopumilaceae* (ASVs 16151 and 15915), which were more abundant in this biotope compared to *Litophyton* and seawater (Supplementary Figures S6A,C). Seawater samples were characterized by higher abundances of *Vibrio* (ASVs 16165, 17698, 16518 and 17430), *Rubritalea* (ASV 16133), *Mycobacterium* (ASV 16161), *Pseudohongiella* (ASV 16692), *Cetobacterium* (ASV 16112), unclassified *Hyphomicrobiaceae* (ASV 16132), and unclassified *Alphaproteobacteria* (ASV 16140) relative to both *Litophyton* and sediments (Supplementary Figure S6B,C). Higher abundances of *Seonamhaeicola* (ASV 17793), unclassified *Nitrosopumilaceae* (ASVs 16151 and 15915), *Woeseia* (ASV 15844) and *Cenarchaeum* (ASV 16125), also distinguished seawater samples from sediments (Supplementary Figure S6C).

In the Red Sea samples, 10 differentially abundant ASVs were found between *Litophyton* and sediments, 47 between *Litophyton* and seawater, and 45 between seawater and sediments (Supplementary Figure S7). *Litophyton* samples were enriched in unclassified *Alphaproteobacteria* (ASVs 17740 and 17741) and BD1-7 Clade (ASV 16741) compared to both sediments and seawater (Supplementary Figures S7A,B). Sediment samples exhibited significantly higher abundances of *Ruegeria* (ASV 17384) and *Candidatus* Nitrosopumilus (ASV 17516) relative to *Litophyton* (Supplementary Figure S7A), and of *Romboutsia* (ASV 116734) and *Candidatus* Nitrosopumilus (ASV 17516) compared to seawater (Supplementary Figure S7C). Seawater samples were characterized by a marked enrichment in *Synechococcus* CC9902 (ASVs 16732 and 16437), *Candidatus* Actinomarina (ASVs 6327 and 690) and *Prochlorococcus* MIT9113 (ASVs 16516 and 693), among others, compared to *Litophyton* (Supplementary Figure S6B). When compared to sediments, seawater samples also exhibited higher abundances of *Synechococcus* CC9902 (ASV 16732), *Prochlorococcus* MIT9113 (ASVs 16516 and 693), and *Candidatus* Actinomarina (ASV 6327) (Supplementary Figure S7C). ASV 16732 (*Synechococcus* CC9902) was also identified among the top 10 contributors to community dissimilarity across all sample groups, as revealed by SIMPER analysis (Figure 4; Supplementary Figure S5).

Detailed results on the comparison of the same biotope across habitats are available in the Extended Results as well as Supplementary Figure S8.

### 3.7 Select ASVs are maintained in octocorals after long-term captivity in aquarium

**Figure 5:**
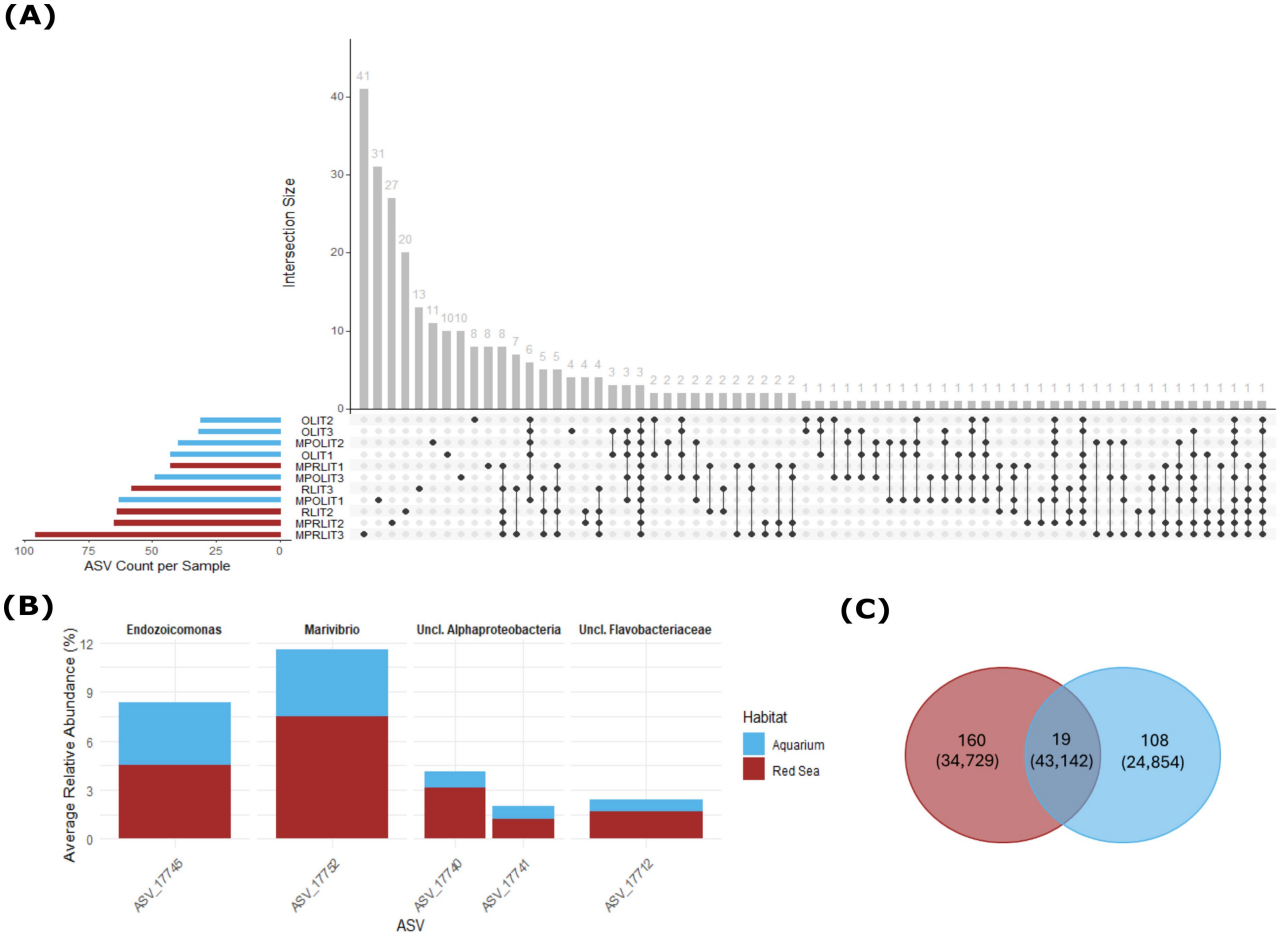
Core prokaryotic ASVs in *Litophyton* samples. Aquarium samples are represented in blue, while Red Sea samples are shown in red. **(A)** Intersection plot illustrating the number of ASVs unique to individual samples (single dots), and shared across multiple samples (connected dots). The horizontal bars (left side) show the total number of ASVs in each *Litophyton* sample. The vertical bars (top) represent the number of ASVs in each intersection. **(B)** Bar chart displaying the relative abundances of the five ASVs present in at least 80% of *Litophyton* samples per habitat. **(C)** Venn diagram showing the number of ASVs detected in *Litophyton* samples from each habitat. An ASV was considered shared if it was present in at least one sample per habitat. Numbers in parentheses indicate the total number of reads associated with ASVs unique to each habitat or shared between them.

Analysis of ASVs shared among *Litophyton* samples across habitats and DNA extraction procedures revealed 19 ASVs present in at least one specimen from each habitat (Figure 5), encompassing diverse bacterial genera and families. Only three ASVs were common to all samples (Figure 5A), namely *Endozoicomonas* ASV 17745, *Marivibrio* ASV 17752 and unclassified *Alphaproteobacteria* ASV 17740, which were more abundant in the Red Sea (4.50, 8.97 and 3.10% average relative abundance, respectively) compared to the aquarium (4.45, 5.90 and 1.03%, respectively).

When the analysis was adjusted to include ASVs present in at least 80% of samples per habitat, two additional ASVs were identified (Figure 5B). Among these, unclassified *Alphaproteobacteria* ASV 17741 exhibited similar relative abundance in the Red Sea samples (1.66% average relative abundance) compared to the aquarium samples (1.13%). Unclassified *Flavobacteriaceae* ASV 17712 was more abundant in the Red Sea (1.70%) compared to the aquarium (0.99%). Further, a total of 19 ASVs were present in at least one sample from each habitat (Figure 5C). Among these, two classified genera, *Marivibrio* and *Endozoicomonas*, were each represented by two distinct ASVs. Despite their limited number, these 19 ASVS comprised 42% of the *Litophyton-*associated reads (43,142 out of 102,725 reads in total), highlighting their ecological relevance. In particular, the genus *Endozoicomonas* contributed with 20,157 reads from two ASVs, ASV 17661—which was present in all Red Sea samples and only in MPOLIT1 from the aquarium, and the previously mentioned ASV 17745. Likewise, when we counted the number of formally classified bacterial families present in at least one *Litophyton* specimen from each habitat, 21 families were found in common among habitats, accounting for 74,542 (72.6%) of the *Litophyton*-associated reads reported in this study.

For detailed results on ASVs shared among sediment samples and among seawater samples across habitats see Extended Results and Supplementary Figures S9 and S10.

### 3.8 Phylogenetic analysis revealed conserved clustering of low abundance *Endozoicomonas* across habitats

**Figure 6:**
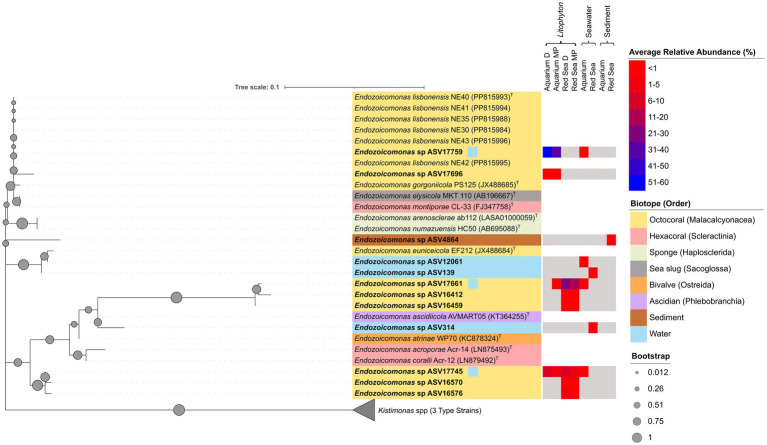
Phylogenetic inference of *Endozoicomonas* ASVs based on the 16S rRNA gene. The tree depicts 12 *Endozoicomonas* Amplicon Sequence Variants (ASVs), all 11 formally described *Endozoicomonas* type strains, and five additional *Endozoicomonas lisbonensis* isolates purified from samples analyzed in this study. Three closely related *Kistimonas* type strains were included as an outgroup. The tree was constructed using the Maximum-Likelihood algorithm with the Kimura 2-parameter model, with a discrete Gamma distribution (8 categories; +G, parameter = 0.3461), and proportion of invariant sites ([+I], 55.84% sites). Partial deletion was used and all positions with less than 85% site coverage were eliminated, resulting in 253 positions in the final dataset. Filled circles indicate bootstrap support based on 1,000 replications. The tree was drawn to scale, with branch lengths representing the number of substitutions per site (scale bar in the figure). Colored shadings indicate the respective biotope. ASVs from this study are highlighted in bold. Accession numbers of the 16S rRNA gene sequences from cultured representatives are provided in parentheses after the strain names. The average relative abundances of each ASV across the different biotopes and habitats analyzed in this study are displayed in the heatmap next to the tree.

The phylogenetic analysis of *Endozoicomonas* ASVs (Figure 6) revealed diverse, so-far uncultured *Endozoicomonas* phylotypes from *Litophyton* specimens and the surrounding seawater (both from the Red Sea and the aquarium) which are phylogenetically distinct from known cultured isolates. One cluster consisted of ASV 17661, which was detected in both aquarium and Red Sea *Litophyton* samples (average relative abundances of 0.09 and 22.24%, respectively) and in aquarium seawater below 1%, together with ASVs 16412 and 16459 from Red Sea *Litophyton*. Pairwise comparisons (Supplementary Table S8) indicated that the closest type strain for this cluster was *E. ascidiicola* AVMART05^T^, although it shared less than 95% 16S rRNA gene homology. Another ASV present in both aquarium (4.45%) and Red Sea (4.50%) *Litophyton* samples, and at low abundance (< 1%) in aquarium seawater, was ASV 17745, clustering with ASVs 16570 and 16576 from Red Sea *Litophyton* samples. The closest type strains to ASV 17745 were *E. gorgoniicola* PS125^T^ and *E. lisbonensis* NE40^T^, both sharing 97.23% sequence similarity. The presence of ASVs 17661 and 17745 in both natural (Red Sea) and artificial (aquarium) *Litophyton* suggests the conservation of a (likely) key symbiont under artificial conditions, indicating that these yet uncultured clades can be maintained under aquarium conditions.

The most abundant *Endozoicomonas* ASV (17759), found enriched in aquarium *Litophyton* samples (46.12%) and present in low abundance in aquarium seawater (< 1%), clustered with the type strain of the recently described species *E. lisbonensis* NE40^T^ (100% similarity) (Marques et al., 2025; Da Silva et al., 2025), which was isolated from the same *Litophyton* specimen OLIT2 that was used in the present study. ASV 17696, found only in aquarium *Litophyton,* was also part of this cluster, sharing 98.81% sequence similarity with *E. lisbonensis* NE40^T^. Notably, at least ASV 17759 (*E. lisbonensis*) represents a culturable, aquarium-specific phylotype that is absent in the Red Sea, but is selectively enriched by *Litophyton* specimens in the aquarium habitat.

Two ASVs, 12061 and 139, identified in aquarium and Red Sea seawater, respectively, exhibited close relatedness to *Endozoicomonas euniceicola* EF212^T^, with sequence similarities above 99%. An ASV detected in Red Sea sediments formed a distinct clade, exhibiting closer, yet only moderate, 16S rRNA gene relationship to *E. gorgoniicola* PS125^T^ and *E. lisbonensis* NE40^T^, with ASV 4864 showing 96.44% similarity to both type strains.

## 4 Discussion

This study compared the prokaryotic communities associated with the tropical octocoral *Litophyton* sp. in a natural Red Sea reef and a large-scale, long-term aquarium system designed to replicate a complex marine environment. We identified consistent core bacterial families—*Endozoicomonadaceae*, *Kiloniellaceae*, and *Spirochaetaceae*, across both habitats, indicating similar octocoral microbiome assembly patterns in two ecologically-independent, natural vs. man-made, settings. Additionally, this study demonstrates that while microbial alpha-diversity in *Litophyton* remained stable across habitats and sample processing methods—direct versus indirect DNA extraction, beta-diversity appeared to be influenced by the sample processing strategy, though interaction effects were not significant and results should be interpreted with caution due to small sample size and dispersion differences. However, the absence of a clear methodological effect when comparing different biotopes within the same habitat suggests that this influence may be context-dependent. Given the comparable alpha-diversity metrics and reliable performance of direct DNA extraction, our findings support this approach as a practical and effective method for coral microbiome studies. Future research should prioritize greater biological replication over parallel extraction strategies to more effectively capture ecologically relevant variation.

Previous microbiome studies comparing aquarium and natural conditions in both octocorals (Bonacolta et al., 2024; Wessels et al., 2017) and hexacorals (Galand et al., 2018; Kooperman et al., 2007; Pratte et al., 2015; Röthig et al., 2017), maintained coral fragments in aquaria with volumes of less than 100 L (when specified), and containing only seawater and the coral fragments studied, to minimize external factors that could influence experimental outcomes. Only the study of Pratte et al. (2015) also included co-occurring coral species. Both Galand et al. (2018) and Pratte et al. (2015) emphasized that timing is critical in these comparisons, as short-term microbiota shifts often stabilize or partially revert to their original states after some time. In contrast, our study focused on octocorals maintained in a well-established, large-scale ecosystem containing approximately 19,000 L of seawater, where the corals have resided for over two decades.

Our analysis demonstrated that long-term captivity within an ecosystem framework preserved unique prokaryotic community features, both in terms of diversity and taxonomic composition, across distinct marine biotopes. These findings are supported by a comprehensive characterization of ASVs and identification of corresponding taxa, which differentiated each biotope in both natural and aquarium settings and remained partially consistent across habitats. This indicates that mitigating the “aquarium effect” (Galand et al., 2018) requires maintaining octocorals within a complex, multi-species ecosystem, thereby potentially preserving their core symbionts.

### 4.1 Preservation of biotope-specific prokaryotic profiles between natural and aquarium habitats

Our findings of distinct prokaryotic communities between biotopes in the wild align with previous studies (Keller-Costa et al., 2017, 2021; Park et al., 2022; Schöttner et al., 2009), which also report higher diversity in sediments and seawater compared to corals. Specifically, we observed a higher prokaryotic richness in sediments, followed by seawater, and a lower richness in *Litophyton* samples. Likewise, Schöttner et al., documented distinct community profiles between cold-water (fjords) hexacorals, seawater and sediments, with bacterial diversity being highest in sediments, followed by seawater, while hexacorals exhibited much lower diversity (Schöttner et al., 2009). Similarly, Park et al. (2022) and Keller-Costa et al. (2017, 2021) found higher bacterial diversity in the surrounding seawater compared with the microbiome of multiple octocoral genera, including *Litophyton*.

Similarly to our findings, when comparing bacterial communities in aquarium settings, Schöttner et al. (2009) and Röthig et al. (2017) found distinct communities between seawater and hexacoral samples after various periods of rearing (3 months and over 1 year, respectively). Notably, Schöttner et al. (2009) observed higher bacterial richness and greater variation in hexacoral samples compared to seawater samples after 3 months of rearing. However, the aquarium conditions in their study were relatively simplified, consisting only of the studied corals and unfiltered natural fjord seawater in a flow-through system, which could explain the observed differences in bacterial richness between coral and seawater samples. To the best of our knowledge, no studies have yet compared the prokaryotic/bacterial communities of aquarium-derived sediments with those of seawater and corals. The identification of distinguishing ASVs enriched in each biotope within aquarium settings and of typical coral-associated and sediment-associated bacteria, respectively, common to each habitat, suggests a likely functional convergence of microbial communities in these biotopes across artificial and natural systems.

Although our study did not find significant differences in ASV richness or Shannon diversity among *Litophyton* specimens from the Red Sea and the aquarium, our results are consistent with those of Kooperman et al. (2007) who observed higher (bacterial) richness and diversity in natural environments compared to aquarium settings. Kooperman et al. (2007) compared the surface mucus microbiota of a hexacoral from the shallow Red Sea (9–24 m) in sterile aquaria and natural environments, finding greater diversity in the latter. Similarly, we noted a slightly higher diversity among *Litophyton* specimens from the Red Sea, even though this difference was not statistically significant. Red Sea *Litophyton* specimens also showed greater variability in prokaryotic community composition which seems to be common among wild corals and has been observed earlier in temperate octocoral species (Keller-Costa et al., 2021).

### 4.2 Long-term captivity in aquarium settings supports the presence of *Endozoicomonadaceae*, *Kiloniellaceae* and *Flavobacteriaceae* symbionts in octocorals

Previous studies on hexacorals have documented changes in bacterial community structure under captive conditions, even though some coral species were able to temporarily maintain their bacterial consortia in sterile aquarium environments (Galand et al., 2018; Pratte et al., 2015; Röthig et al., 2017). Our findings, however, suggest a higher degree of microbiome resemblance in *Litophyton* sp., with multiple bacterial taxa consistently retained across both habitats (Red Sea vs. aquarium). This is supported by the relatively low *F*-value (1.11) and *R*^2^ (0.10) from the PERMANOVA analysis, indicating that host-associated prokaryotic communities were more conserved across habitats than those of seawater and sediments, which displayed greater compositional variability. Across all *Litophyton* specimens, the class *Gammaproteobacteria* typically dominated, with *Alphaproteobacteria*, *Spirochaetia* and *Epsilonproteobacteria* being also common. *Gammaproteobacteria*, *Alphaproteobacteria* and *Epsilonproteobacteria* are among the dominant bacterial classes of Red Sea corals (reviewed in Delgadillo-Ordoñez et al., 2022) and corals in general (e.g., Lawler et al., 2016; Liu et al., 2024; Prioux et al., 2024; Quintanilla et al., 2022; van de Water et al., 2018a).

The higher relative abundance of *Campylobacterales* (class *Epsilonproteobacteria*) in aquarium-kept *Litophyton* specimens aligns with previous reports of this order in various octocoral genera (Kellogg et al., 2016; Lawler et al., 2016; van de Water et al., 2024; Weiler et al., 2018). Members of the order *Campylobacterales* have also been detected in diseased marine organisms, including oysters (Lokmer and Mathias Wegner, 2015), sponges (Fan et al., 2013), octocorals (Vezzulli et al., 2013), and various hexacoral genera (Daniels et al., 2015; Frias-Lopez et al., 2002; Soffer et al., 2015; Sunagawa et al., 2009), raising questions about their roles and positioning along the mutualism–parasitism continuum. Although this order is functionally diverse and taxonomically broad, some members are hypothesized to contribute to nitrogen cycling, particularly through nitrate or nitrite ammonification pathways (Kellogg et al., 2016). Their association with both healthy and stressed hosts suggests potential functional plasticity or opportunistic behavior, which warrants further investigation.

Our results revealed the retention of multiple, typical symbiont taxa in *Litophyton* even after decades in captivity. Even though interindividual variability between coral samples from natural environments is a well-documented phenomenon (reviewed in Maher et al., 2022), the family *Endozoicomonadaceae* was present in all *Litophyton* specimens in both habitats, dominating the aquarium specimens and one out of three Red Sea specimens. Notably, the families *Kiloniellaceae* and *Spirochaetaceae* were consistently detected across all *Litophyton* specimens examined in this study. Both families were identified in both aquarium and natural settings, although *Spirochaetaceae* did not share ASVs between habitats.

The dominance of *Endozoicomonas* (*Endozoicomonadaceae*, *Gammaproteobacteria*) and *Spirochaeta* (*Spirochaetaceae*, *Spirochaetia*) has been well-documented across tropical, temperate, and cold-water octocorals, regardless of depth, and these taxa have been identified as key components of the microbiome of several healthy octocoral species (Bayer et al., 2013; Bonacolta et al., 2024; Cleary et al., 2020, 2021; Gray et al., 2011; Haydon et al., 2022; Holm and Heidelberg, 2016; Jahajeeah et al., 2023; Keller-Costa et al., 2021, 2022; Kellogg et al., 2016; Kellogg and Pratte, 2021; Lawler et al., 2016; McCauley et al., 2020; Monti et al., 2023; O’Brien et al., 2020; Park et al., 2022; Prioux et al., 2024; Steinum et al., 2024; Tignat-Perrier et al., 2022, 2023; van de Water et al., 2016, 2017, 2018b, 2020; Vohsen and Herrera, 2024; Weiler et al., 2018).

The genus *Endozoicomonas* is particularly abundant in tropical octocorals (Cleary et al., 2020, 2021; Easson et al., 2024; Haydon et al., 2022; Mccauley et al., 2016; McCauley et al., 2020; Monti et al., 2023; O’Brien et al., 2021; Park et al., 2022; Wessels et al., 2017), and has been found to be preserved in hexacoral microbiomes maintained for up to 6 months under sterile aquarium conditions (Galand et al., 2018). However, contrastingly to our findings, studies have shown *Endozoicomonas* to be partially or entirely lost during hexacoral long-term captivity exceeding 2 years (Barreto et al., 2021; Puntin et al., 2024). Previous studies suggest that the genus *Endozoicomonas* plays multiple roles in marine invertebrates, particularly within coral holobionts. A key function proposed for this genus, and other members of the *Endozoicomonadaceae* family, is polysaccharide degradation. Genomic and metagenome-assembled genome analyses revealed that these bacteria are likely to hydrolyze chitin, thereby contributing to carbon and nitrogen cycling (da Silva et al., 2023; Keller-Costa et al., 2022). Beyond chitin and carbohydrate degradation in general, these symbionts have been implicated in several metabolic processes, including the synthesis of amino acids and B vitamins (Jensen et al., 2021; Keller-Costa et al., 2022; Neave et al., 2017; Pogoreutz et al., 2022), nitrogen metabolism (Neave et al., 2014, 2016), and sulfur cycling via the breakdown of dimethylsulfoniopropionate (DMSP) and dimethyl sulfide (DMS) (Neave et al., 2017; Raina et al., 2009; Sweet et al., 2021; Tandon et al., 2020). Several of the proposed metabolic roles are inferred from the analyses of so-far unculturable *Endozoicomonadaceae* lineages, which is the case of the ASVs shared between *Litophyton* specimens in the Red Sea and the aquarium.

The *Spirochaetaceae* family is known for its widespread presence across various regions and depths in diverse marine environments. This family is frequently reported as an abundant member of the tropical octocoral microbiome (Cleary et al., 2020, 2021; Haydon et al., 2022; O’Brien et al., 2020, 2021; Park et al., 2022; Wessels et al., 2017). It has also been identified among the taxa maintained in hexacorals reared under simplified aquarium conditions (only corals and seawater) for over 6–12 months (Galand et al., 2018; Röthig et al., 2017). Despite their widespread distribution in octocorals, the functional roles of this family within the coral holobiont remain largely undetermined. Curiously, a potential association between coral colony coloration and differential abundances of *Spirochaeta* has been proposed in *Corallium rubrum*, a Mediterranean octocoral species (van de Water et al., 2024).

The identification of *Kiloniellaceae*, although rarely reported, is not unprecedented. This family has recently been documented in the microbiome of tropical and cold-water octocorals (Estaque et al., 2024; Monti et al., 2023), as well as among the dominant bacterial taxa associated with cold-water anemones (Quintanilla et al., 2022), and cultured representatives exist from the octocoral *Eunicella labiata* (Keller-Costa et al., 2017).

Although research on the prokaryotic communities of *Litophyton* is scarce, previous studies have highlighted the dominance of *Endozoicomonas* and *Cellvibrionaceae* (*Gammaproteobacteria*), as well as the presence of *Spirochaeta*, among other taxa (Alsharif et al., 2023; Park et al., 2022). The identification of taxa in aquarium-kept *Litophyton* in this study aligns with these findings, indicating that at least part of the octocoral microbiome can be preserved long-term in controlled aquarium ecosystems, and that the microbial community structure remains broadly comparable to that observed in wild populations.

While our study focused on the core microbiome, aquarium conditions, and DNA sampling strategies, we acknowledge that a wide range of additional factors influence the stability and composition of coral-associated microbial communities. Environmental variables such as temperature, pH, salinity, nutrient availability and oxygen levels, can fluctuate naturally on diel and seasonal scales, contributing to both temporal variability and adaptive microbial responses (Cannon et al., 2021; Haydon et al., 2022; Steinum et al., 2024). Geographic location and depth further shape the microbiome of octocorals (Osman et al., 2020; Vohsen and Herrera, 2024). Moreover, the coral holobiont comprises multiple niches, including the mucus layer and tissue, that host functionally diverse and compositionally distinct microbial communities (van de Water et al., 2024; Weiler et al., 2018). These compartment-specific dynamics add another layer of complexity to microbiome stability. To fully understand the scope and limitations of microbiome preservation, future studies should investigate additional coral taxa, incorporate longitudinal sampling across diverse climatic regions, and consider ecological parameters such as depth gradients and compartment-specific sampling. By addressing these variables, future research can better delineate the extent to which aquaria can function as partial reservoirs of native coral microbiomes and clarify the environmental and biological boundaries of microbial stability.

### 4.3 Sediments of natural and artificial habitats share *Nitrosopumilaceae*, *Woeseiaceae*, *Pirellulaceae* and *Flavobacteriaceae* spp

Similarly to octocoral, the sediments biotope exhibited similar bacterial taxa across habitats. All sediment samples shared a diverse array of low-abundance taxa that spanned *Acidimicrobiia*, *Alphaproteobacteria*, *Gammaproteobacteria*, *Nitrososphaeria*, and *Plantomycetes*, among others.

The archaeal family *Nitrosopumilaceae* (*Nitrososphaeria*) has been increasingly reported in association with sediments, corals, sponges, and ascidians (Begmatov et al., 2021; Keller-Costa et al., 2017; Moreno-Pino et al., 2020; O’Brien et al., 2020; Robbins et al., 2021; Stroeva et al., 2023; Zhang et al., 2019). In this study, members of the genus *Candidatus* Nitrosopumilus (*Nitrosopumilaceae*) were identified in both habitats. This genus is characterized by its ability to oxidize ammonia, playing a crucial role in nitrogen cycling (Begmatov et al., 2021; Engelberts et al., 2020; Könneke et al., 2005; Robbins et al., 2021). Furthermore, genomic analyses of *Nitrosopumilaceae* associated with corals and marine sponges have suggested additional metabolic capabilities, including carbon fixation via the 3-hydroxypropionate—4-hydroxybutyrate (HP-HB) cycle (Engelberts et al., 2020; Moreno-Pino et al., 2020). The *Pirellulaceae* family (*Planctomycetia*) is widely distributed across multiple marine settings, including sediments from diverse climates (Markovski et al., 2022; Vitorino et al., 2022) and macroalgae (e.g., Wiegand et al., 2021; reviewed in Vitorino and Lage, 2022). Members of this family have demonstrated antimicrobial activity (Vitorino et al., 2022), although research on *Pirellulaceae* remains limited. The *Woeseiaceae* family (*Gammaproteobacteria*), also referred to as the JTB255 bacterial group, is a ubiquitous and highly abundant taxon in marine sediments, being considered as a core component of the global marine sediment microbiota (Begmatov et al., 2021; Bienhold et al., 2016; Chen et al., 2021, 2024; Dyksma et al., 2016; Hoffmann et al., 2020; Markovski et al., 2022; Mußmann et al., 2017; Rincón-Tomás et al., 2024; Stroeva et al., 2023; Zhou et al., 2022). Genes involved in carbon, hydrogen and sulfur metabolism have been identified for members of this group, however, no functional activities have been experimentally ascertained to date (Dyksma et al., 2016; Hoffmann et al., 2020; Mußmann et al., 2017). The *Flavobacteriaceae* family is commonly associated with diverse marine environments (summarized in Hu et al., 2020; Silva et al., 2023), with multiple reports documenting the isolation of members of this family from marine sediments (Chen et al., 2021, 2024; Markovski et al., 2022). Members of the *Flavobacteriaceae* family are proficient polysaccharide degraders and have been suggested to play a role in nitrogen and carbon cycling (Chen et al., 2024; Silva et al., 2019).

### 4.4 Divergent seawater microbiomes

The seawater biotope presented the most contrasting microbiomes across habitats from the present study. Nonetheless, the *Flavobacteriaceae* (*Bacteroidia*), *Vibrionaceae* (*Gammaproteobacteria*) and *Nitrosopumilaceae* (*Nitrososphaeria*) families were identified as common across habitats, along with the genera *Romboutsia* (*Peptostreptococcaceae*, *Clostridia*) and *Vibrio* (*Vibrionaceae*, *Gammaproteobacteria*).

*Flavobacteriaceae* was among the most abundant bacterial families in the seawater of coral-rearing aquaria (Röthig et al., 2017) and was also commonly observed in natural seawater and healthy tropical Red Sea coral microbiomes (Delgadillo-Ordoñez et al., 2022). In this study, *Vibrionaceae* ASVs were enriched in aquarium seawater compared to the reef habitat. While the *Vibrionaceae* family is extensively dispersed across marine ecosystems and frequently reported in healthy coral microbiomes (Bally and Garrabou, 2007; Daniels et al., 2015; Delgadillo-Ordoñez et al., 2022; Martin et al., 2002; Prioux et al., 2023; Rubio-Portillo et al., 2018; Sun et al., 2023; Sweet et al., 2021; Vezzulli et al., 2013; Weiler et al., 2018), some members of this family are considered opportunistic or potentially pathogenic, often linked to coral injuries and diseases (Daniels et al., 2015; Gignoux-Wolfsohn and Vollmer, 2015; Keller-Costa et al., 2021, 2022; Pollock et al., 2017; Rubio-Portillo et al., 2018; Sweet et al., 2019; Ziegler et al., 2016). Previous research demonstrated that natural seawater samples from *Litophyton* collection sites were dominated by *Vibrio* spp. (Park et al., 2022), reinforcing the role of seawater as a potential reservoir for coral-associated bacteria. In addition to being identified in sediment samples (as discussed above), *Nitrosopumilaceae* were also detected in aquarium and Red Sea seawater samples, highlighting their broader distribution across biotopes. Members of the *Romboutsia* genus are obligate anaerobic bacteria isolated from diverse environments, including aquatic habitats, the gastrointestinal tracts of fishes, humans, chickens, and rats, as well as coastal estuarine mud and alkaline-saline lake sediments (Gerritsen et al., 2014; Jurburg et al., 2019; Maheux et al., 2017a, 2017b; Ricaboni et al., 2016; Wang et al., 2015; Wu et al., 2020). Some species have demonstrated probiotic activities particularly in association with rat intestines (Gerritsen et al., 2017). Genomic analyses have revealed potential roles in carbohydrate metabolism and nitrogen fixation (Fan et al., 2024; Gerritsen et al., 2019). In our aquarium setting—home to multiple fish species, sea cucumbers, snails, hermit crabs, and both hexacoral and octocoral taxa—fish gut communities represent a plausible source for *Romboutsia* detected in seawater samples. Moreover, the family *Peptostreptococcaceae*, to which *Romboutsia* belongs, has been commonly identified in the Red Sea corals (Delgadillo-Ordoñez et al., 2022).

The seawater from the aquarium is artificial and thus a biotope that is likely more impacted by the animals it houses, and aquarium management practices such as regular cleaning, feeding (of living organisms in the tank), and continuous water recirculation and monitoring. The divergence between natural and aquarium seawater prokaryotic communities likely reflects these practices, together with key parameters such as salinity, pH, nutrient concentrations, dissolved oxygen, temperature, and light, which are tightly controlled and stabilized in the aquarium, in contrast to the variability found in natural environments. Although the artificial aquarium seawater is not sterile, the microbial load is low as evidenced by very low DNA quantities (< 0.2 ng/μL) compared to an average of 2.4 ng/μL in Red Sea seawater samples. The combination of low microbial abundance, continuous water filtration and treatment, and regular renewal likely prevents the establishment of a stable seawater microbial community, which may explain the higher abundance of potentially opportunistic bacteria in this biotope.

### 4.5 *Endozoicomonadaceae* may reflect both the persistence of natural symbionts and microorganisms acquired in captivity

Given the literature surrounding *Endozoicomonas* spp. as core symbionts of corals, we aimed to explore the phylogenetic affiliation of the ASVs identified in this study in greater detail. The separate clustering of most ASVs by biotope aligns with reports of co-phylogeny between this genus/family and corals (da Silva et al., 2023; Keller-Costa et al., 2017, 2022; O’Brien et al., 2021; Pollock et al., 2018; Prioux et al., 2024). The clustering of most *Litophyton*-associated ASVs near *E. gorgoniicola* and *E. lisbonensis*, two species isolated from tropical octocorals (Pike et al., 2013; Da Silva et al., 2025), may reinforce these biotope-specific associations. Moreover, the relatively low sequence similarity (typically below 98%) between ASVs common to octocorals across different habitats, when compared to cultured type strains, suggests that these preserved ASVs, together with ASVs detected only in natural biotopes, are likely to represent yet-uncultivated *Endozoicomonas* lineages. This aligns with the well-documented challenge of cultivating members of the widespread *Endozoicomonadaceae* family, despite the successful isolation of several strains. Nonetheless, we acknowledge that intra-genomic variation in 16S rRNA gene copies within a single strain may confound ASV clustering. As such, finer-scale resolution would require multi-locus or whole-genome approaches.

The most abundant ASV from the aquarium *Litophyton* aligned with isolates cultured from the same *Litophyton* samples by Marques et al. (2025), strongly suggesting they represent the same organism, recently described as the novel species *E. lisbonensis* (Da Silva et al., 2025). This novel *Endozoicomonas* species is a facultative anaerobe, able to reduce nitrates to nitrates, produce siderophores to chelate iron from the environment, and to degrade a range of polysaccharides such as chitin, cellulose, and xylan, underscoring its metabolic versatility (Da Silva et al., 2025).

The identification of both shared ASVs and ASVs unique to the aquarium habitat in the genus *Endozoicomonas* suggests that the microbiomes of captive octocorals may result from a combination of core symbionts retained from natural populations and microorganisms potentially acquired from the rearing system. For instance, ASVs 17661 and 17745, detected in both wild and aquarium *Litophyton* specimens, may represent core symbionts that were retained following transplantation to the aquarium. Since aquarium corals are primarily propagated vegetatively, this mode of reproduction likely facilitates the consistent transmission of associated microbial communities across generations. However, vertical transmission, typical of sexual reproduction in wild populations, may also contribute to the persistence of specific microbial taxa, including *Endozoicomonadaceae*. Vertical transmission is well-documented in coral-microbe associations (Bernasconi et al., 2019; Damjanovic et al., 2020; Maire et al., 2024) and is especially evident for the *Endozoicomonadaceae* family in brooding species (Damjanovic et al., 2020; Maire et al., 2024), which includes *Litophyton* (Benayahu et al., 1990). The case of *Endozoicomonas lisbonensis* (ASV 17759), however, is less straightforward. Although its presence in aquarium seawater raises the possibility of horizontal acquisition from the surrounding environment followed by enrichment in *Litophyton*, this does not exclude the alternative scenario that it was already associated with the *Litophyton* specimens *in situ* and co-transplanted to the aquarium—with subsequent secondary release into the surrounding seawater. However, without detailed information on the origin of the *Litophyton* specimens or on other potential hosts within the aquarium, the exact route of acquisition remains unclear. Future studies integrating co-phylogenetic analyses between *E. lisbonensis* and more *Litophyton* specimens and species from different marine environments and geographic regions, along with expanded microbial surveys of the aquarium environment and its animal inhabitants, will be essential to elucidate the nature of this association.

While the beneficial role of *Endozoicomonas* in coral health remains under debate (Pogoreutz and Ziegler, 2024), its dominance in healthy octocorals is well-documented (Keller-Costa et al., 2021, 2022; van de Water et al., 2018a). The here-found persistence of this genus in aquarium settings further underscores its resilience, and potential importance in maintaining coral health.

### 4.6 Concluding remarks

This study compared the microbiomes of the octocoral *Litophyton*, seawater, and sediments from a long-term aquarium designed to mimic a natural coral reef ecosystem. Our findings revealed that each biotope maintained distinct microbiomes, demonstrating that the “aquarium effect,” associated with microbiome homogenization (Galand et al., 2018), can be mitigated in controlled large-scale mesocosms resembling complex ecosystems. Shared taxa—particularly of the *Endozoicomadaceae* and *Spirochaetaceae* families—suggest that key components of natural microbiomes were preserved in octocorals even after more than 25 years of captivity. Our results indicate aquaria as repositories of healthy host-associated microbiomes, capable of safeguarding both host and microbial diversity. Our study reveals a previously unnoticed role of large-scale aquarium ecosystems in microbiome stewardship and highlights their potential as sources of microbiota for microbiome-engineering–based reef restoration efforts.

Future research should explore whether the here observed microbiome preservation extends to other coral taxa and examine the specific functional capacities of the microbial taxa retained. Additionally, understanding the mechanisms that support the maintenance of natural microbiomes in aquaria could guide best practices for coral reef conservation and management, including the development of advanced aquarium systems for microbiome-based restoration strategies.

## Data Availability

The raw sequence reads of the 16S rRNA gene amplicon dataset of this study can be found in the Sequence Read Archive (SRA-NCBI) under the BioProject accession number PRJNA1256069 and BioSample accession numbers [SAMN48159183 –SAMN48159208]. The mitochondrial cytochrome c oxidase subunit I and mutS Sanger sequences of the *Litophyton* specimens are deposited at NCBI GenBank under accession numbers [PV573986–PV573991] and [PV568289–PV568294], respectively.

## Footnotes

^1^ https://earthmicrobiome.org/protocols-and-standards/16s/
