# Supplementary material for: Selective shaping of prokaryotic communities and core symbiont maintenance suggest large-scale aquarium facilities as reservoirs of microbiome diversity in octocorals
Source: Front Microbiol. 2025 Sep 3;16:1651109. doi: 10.3389/fmicb.2025.1651109 (PMC12442434; doi:10.3389/fmicb.2025.1651109)
Supplement: Supplementary file 2 [file Supplementary_file_2.docx]

**Supplementary Material – File 2**

**Title**

Selective shaping of prokaryotic communities and core symbiont maintenance suggest large-scale aquarium facilities as reservoirs of microbiome diversity in octocorals

**Authors**

Matilde Marques^1,2^, Francisco Pascoal^3,4^, Helena Villela^5,^ Elsa Santos^6^, Núria Baylina^6^, Raquel S. Peixoto^5^, Tina Keller-Costa^1,2#^ & Rodrigo Costa^1,2#^

**Affiliations**

^1^ Institute for Bioengineering and Biosciences (iBB) and Institute for Health and Bioeconomy (i4HB), Instituto Superior Técnico (IST), University of Lisbon, Lisbon, Portugal

^2^ Department of Bioengineering, Instituto Superior Técnico (IST), University of Lisbon, Lisbon, Portugal

^3^ Interdisciplinary Centre of Marine and Environmental Research (CIIMAR), University of Porto, Terminal de Cruzeiros do Porto de Leixões, Av. General Norton de Matos s/n, 4450-208 Porto, Portugal

^4^ Departamento de Biologia, Faculdade de Ciências, Universidade do Porto, rua do Campo Alegre s/n, 4169– 007 Porto, Portugal

^5^ Division of Biological and Environmental Science and Engineering (BESE), King Abdullah University of Science and Technology (KAUST), Thuwal, Saudi Arabia

^6^ Oceanário de Lisboa, Esplanada D. Carlos I, Lisbon, Portugal

**^#^Corresponding authors:** Rodrigo Costa ([rodrigoscosta@tecnico.ulisboa.pt](mailto:rodrigoscosta@tecnico.ulisboa.pt)) and Tina Keller-Costa ([tinakellercosta@tecnico.ulisboa.pt](mailto:tinakellercosta@tecnico.ulisboa.pt)).

**Detailed Methodology**

**Octocoral phylogenetic inference**

To ascertain the genus-level taxonomy of the sampled octocoral specimens and perform molecular barcoding assessments, the TC-DNA from each sample was subjected to PCR amplification of the mitochondrial marker genes Mutator S (mtMutS; formerly msh1) and Cytochrome Oxidase Subunit I (COI). The mtMutS gene is a mitochondrial mismatch repair gene specific to octocorals and serves as a reliable genetic marker for identifying octocorals (McFadden et al., 2010). For this gene, a target region of c.a. 870 bp was amplified using the primers ND42599F (5'-GCCATTATGGTTAACTATTAC-3'; (France and Hoover, 2002)) and MUT3458R (5'-TSGAGCAAAAGCCACTCC-3'; (Sánchez et al., 2003)). The COI primer pair targets the mitochondrial cytochrome C oxidase subunit I, with an expected fragment size of 1,000 bp. Amplification was performed using the primers COII8068F (5'-CCATAACAGGACTAGCAGCATC-3'; (McFadden et al., 2004)) and COIOCTR (5’-ATCATAGCATAGACCATACC-3’; (McFadden et al., 2010)). PCR reaction mixtures were prepared as detailed in (Esteves et al., 2013). Thermal cycling started with an initial denaturation step of 94 °C for 5 min, 40 cycles of 94 °C for 30 s, 58 °C for 90 s, 72 °C for 60 s and a final extension step of 72 °C for 10 min. PCR products were cleaned with Sephadex G50 (GE Healthcare Bio-Science AB, Uppsala, Sweden) columns, quantified with an Invitrogen Qubit 4 (Fisher Scientific) and the Qubit dsDNA BR assay kit, and subjected to Sanger sequencing using the respective forward primer. All sequences were manually trimmed using BioEdit v7.0.5.3 (Hall, 1999). For phylogenetic inference, voucher sequences for each gene (19 sequences for COI and 12 sequences for mtMutS) were retrieved from NCBI on December 20^th^, 2024. Three groups of keywords were used with the restrictor “AND” between them, and the restrictor “OR” for the search within each group. One group had the search item “voucher”. The second group, depending on the targeted gene, consisted either of “MutS” and “msh1” for the mtMutS gene, or of “COI” and “cox1” for the COI gene. The last group included all synonymized genus names attributed to *Litophyton* octocorals (based on (WoRMS Editorial Board, 2024): “*Litophyton*”, “*Ammicella*”, “*Lithophyton*”, “*Lithophytum*”, “*Nephthea*”, “*Nephthya*” and “*Nephtya*”. All sequences, six sequences from the here studied samples for each gene, along with 19 voucher sequences for COI and 12 voucher sequences for mtMutS, were aligned using the ClustalW algorithm in the MEGA version X (Kumar et al., 2018). The optimal evolutionary model was determined using the “find best DNA/Protein Model” function for each gene. For both genes, maximum-likelihood trees the Neighbour-Joining (NJ) and BioNJ algorithms were constructed using the Tamura 3-parameter model with uniform rates and 1,000 bootstrap repetitions. Positions with less than 85% site coverage (allowing < 15% gaps, missing data, or ambiguous bases) were eliminated using partial deletion.

**Extended Results**

**Distinct ASV abundance distributions across biotopes and habitats underpin biotope-specific assembly of prokaryotic communities**

When comparing the same biotope across habitats, five differentially abundant ASVs were identified for *Litophyton*, 47 for seawater and 107 for sediments (Supplementary Material, Figure S8). *Litophyton* samples from the aquarium were characterized by higher abundances of *Endozoicomonas* (ASV 17759) and unclassified *Campylobacterales* (ASV 17762) (Figure S8C). In contrast, the Red Sea-derived *Litophyton* samples were slightly more enriched in unclassified *Alphaproteobacteria* (ASV 17740), an ASV significantly enriched (*p*-value < 0.05) in *Litophyton* samples from both habitats in comparison to the other biotopes (Figure S6A, B; Figure S7A, B), and BD1-7 clade (ASV 16741). An enrichment of several ASVs from the *Cyanobacteriota* phylum, such as *Synechococcus* CC9902 (ASV 16732) and *Prochlorococcus* MIT9313 (ASVs 16516 and 693) was observed in the Red Sea in comparison with aquarium seawater (Figure S8B). Conversely, aquarium seawater samples were enriched in opportunistic bacterial groups such as *Vibrio* (ASVs 16165, 17698 and 16518) and *Enterovibrio* (ASV 16158) in comparison with Red Sea samples. Sediment samples from the aquarium were enriched in taxa such as *Woeseia* (ASVs 15844, 11277, 11270, 15958, 11096), unclassified *Fibrobacteraceae* (ASV 177780), *Cenarchaeum* (ASVs 16036 and 16125), unclassified *Nitrosopumilaceae* (ASVs 16151, 11279 and 15915) and *Candidatus* Nitrosopumilus (ASVs 12977, 16142, 14516 and 16094) (Figure S8A). In contrast, sediment samples from the Red Sea were characterized by higher abundances of *Ruegeria* (ASV 17384) and *Romboutsia* (ASV 16734).

**Select ASVs are maintained in octocorals after long-term captivity in aquarium**

The analysis of ASVs shared among seawater samples across habitats identified four ASVs common to all samples (Supplementary Material, Figure S9A). Broadening the scope to include ASVs present in at least 65% of the samples (i.e.: at least two out of three per habitat), the number of shared ASVs increased to seventeen. The ASVs consistently shared across the six seawater samples were assigned to Clade la, *Romboutsia*, *Vibrio* and *Candidatus* Nitrosopumilus (one ASV each). The extended core microbiome comprised additional ASVs classified as *Cetobacterium*, *Clostridium*, *Epulopiscium,* *Photobacterium*, *Pseudoalteromonas*, *Psychrobacter*, *Qipengyuania*, *Shewanella*, *Staphylococcus*, *Turicibacter*, and unclassified *Alphaproteobacteria* (one ASV per taxon); and three ASVs classified as *Vibrio* (Figure S9B). Most ASVs were more abundant in the aquarium habitat, with their relative abundances overall not exceeding 2% per habitat. Conversely, *Epulopiscium* (ASV 16712), *Romboutsia* (ASV 16734), *Turicibacter* (ASV 16350), and unclassified *Alphaproteobacteria* (ASV 17741) were more abundant in the Red Sea.

The number of ASVs shared between all sediment samples from both the aquarium and the Red Sea was notably higher, totalling 68 ASVs (Supplementary Material, Figure S10A). The average relative abundance of each ASV within each habitat remained consistently below 1% (Figure 10B), except for *Candidatus* Nitrosopumilus ASV 17516 which presented an average relative abundance of 2.97% in aquarium samples, and of 0.58% in Red Sea samples. Shared taxa included six ASVs for *Candidatus* Nitrosopumilus; three for *Woeseia*; and two for *Epulopiscium*, *Ilumatobacter*, *Mariniblastus*, *Rubripirellula*, unclassified *Hyphomicrobiaceae*, unclassified *Microtrichaceae*, unclassified *Nitrosopumilaceae,* unclassified *Pirellulaceae,* unclassified *Actinomarinales*, unclassified *Thalassocaculales*, unclassified BD2-11 terrestrial group, and unclassified *Gammaproteobacteria*. ASV abundances were usually evenly distributed between the two habitats. However, aquarium sediment samples were more enriched in taxa such as *Candidatus* Nitrosopumilus, *Cenarchaeum, Bythopirellula*, Planctobacterium, unclassified D90, unclassified *Nitrosopumilaceae*, and *Vibrio,* whereas Red Sea sediment samples exhibited higher relative abundances of *Epulopiscium, Hoeflea*, *Romboutsia*, *Rubripirellula*, *Ruegeria*, Sva0996 marine group, *Thalassocola,* and *Zeaxanthimibacter* (Figure S10B).

**Supplementary Figures**

**Figure S1. Rarefaction curves based on sequencing depth.** (A) Rarefaction curves for all 24 samples; (B) Rarefaction curves for the 12 Litophyton sp. samples only. Samples originate from the aquarium: Litophyton (direct DNA extraction: OLIT1–OLIT3; microbial pellets: MPOLIT1-MPOLIT3), artificial seawater (OASW1–OASW3), and sediments (OSED1–OSED3), as well as from the Red Sea: Litophyton (direct extraction: RLIT1–RLIT3; microbial pellets: MPRLIT1-MPRLIT3), surrounding seawater (RSW1–RSW3), and sediments (RSED1–RSED3).


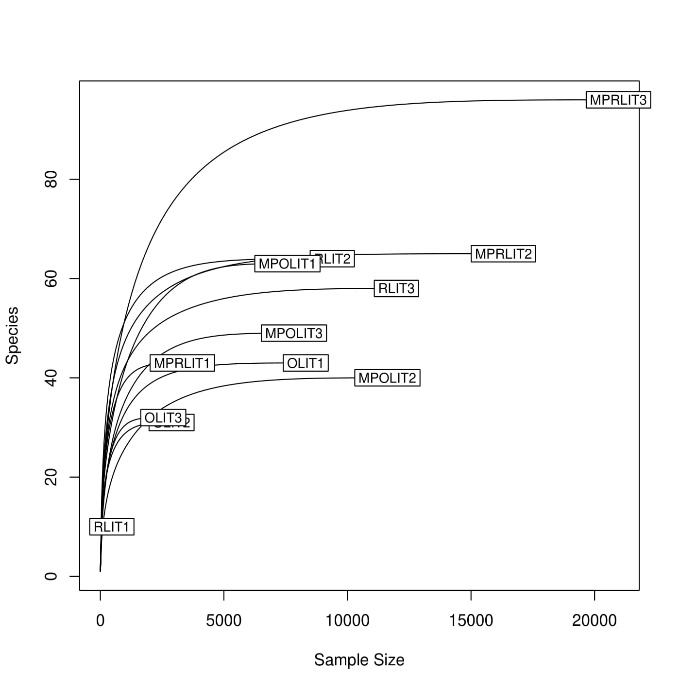

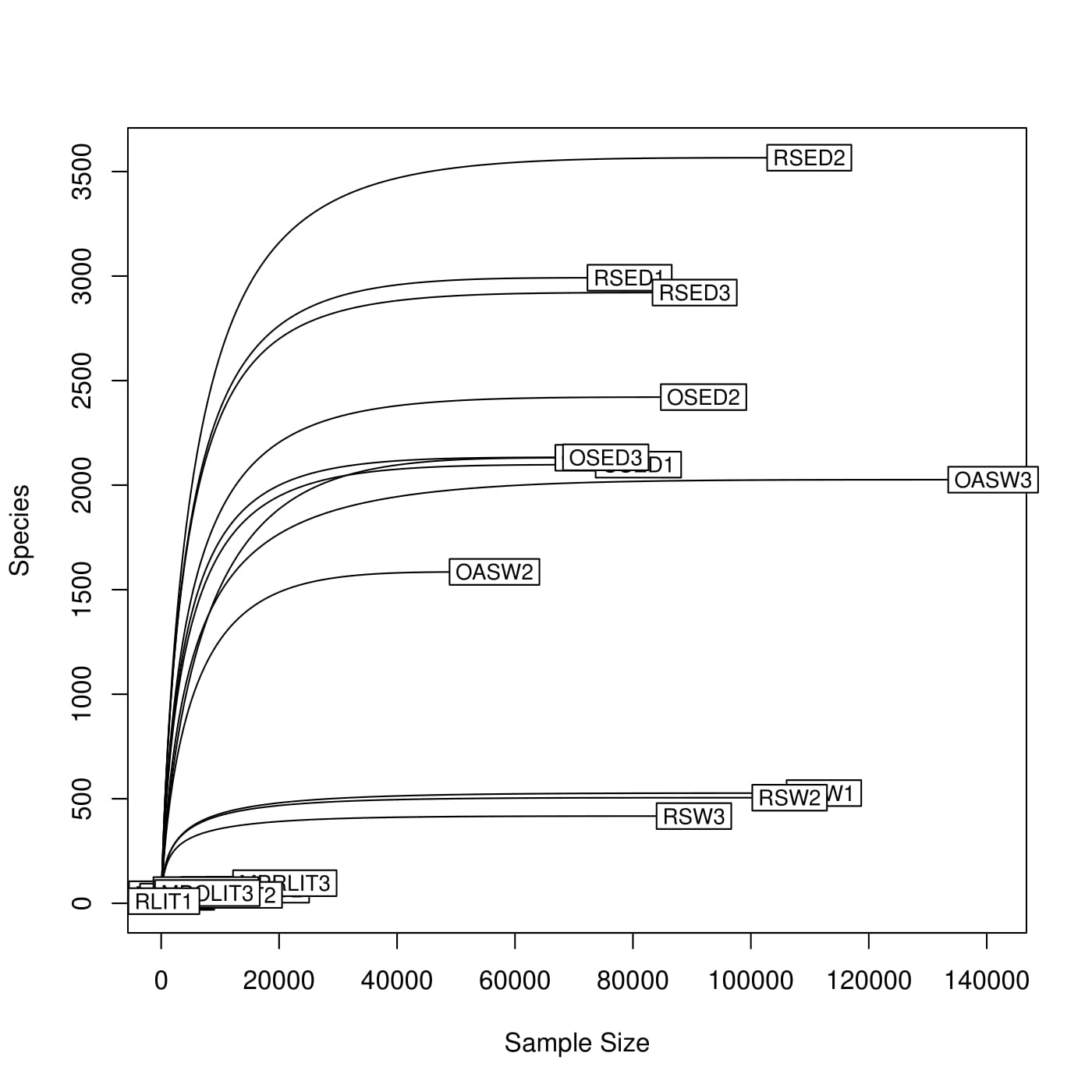


**(A)**

**(B)**


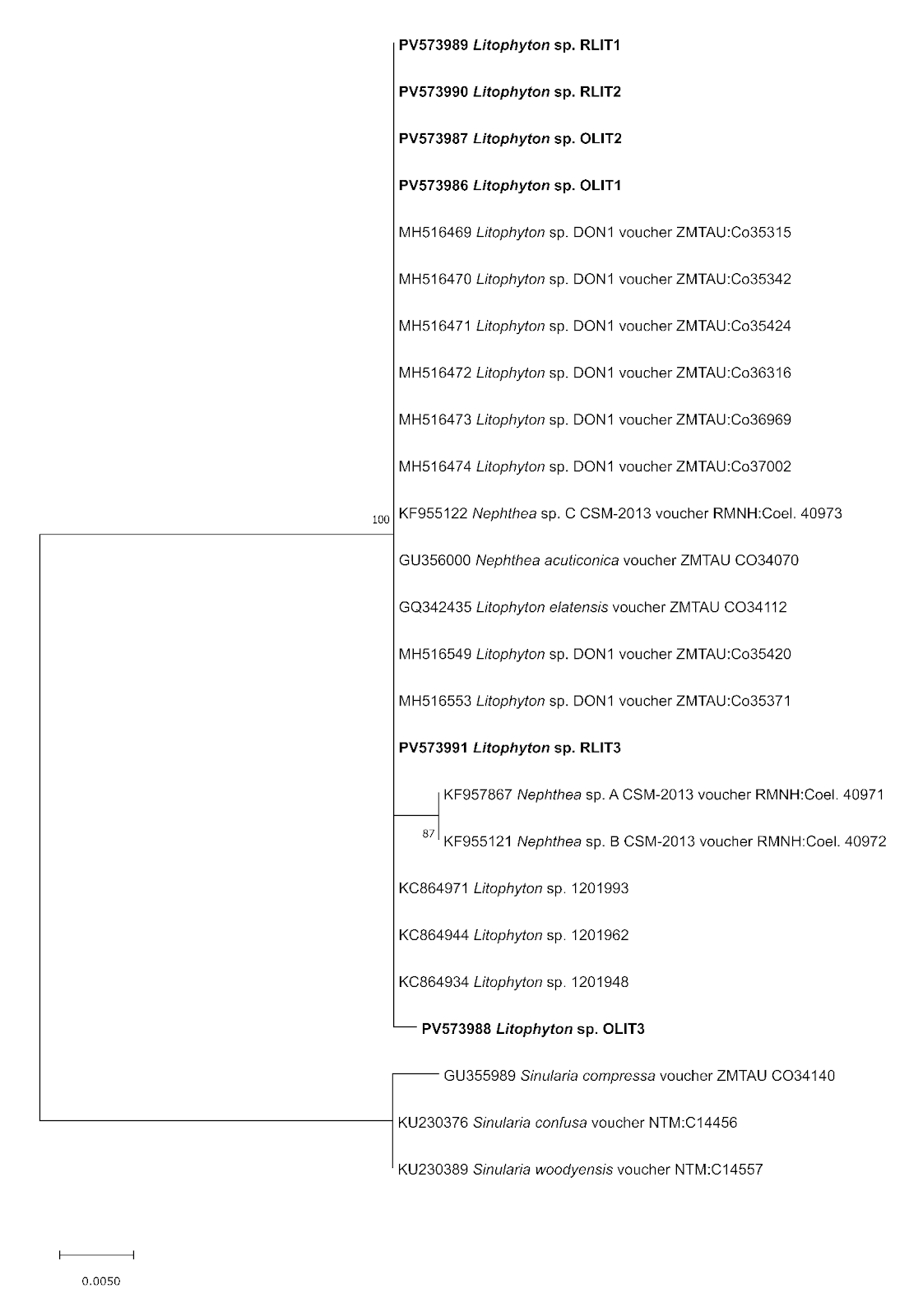


**Figure S2. Phylogenetic inference, based on the cytochrome c oxidase subunit I (COI/COX) gene, of the octocoral samples used in this study.** The tree depicts six octocoral COI gene sequences from the samples here studied (OLIT1-OLIT3 from aquarium corals and RLIT1-RLIT3 from Red Sea corals, all in bold), and sixteen voucher COI gene sequences of the genus Litophyton (formerly Nephthea). Moreover, three voucher COI gene sequences of the genus Sclerophytum (formerly Sinularia) were used as an outgroup to root the tree. The voucher sequences were publicly available and retrieved from Genbank, NCBI. The tree was constructed using the Maximum-Likelihood algorithm with the Tamura 3-parameter model, with uniform rates and 1,000 bootstrap repetitions. Codon positions included were 1st+2nd+3rd+Noncoding. Partial deletion was used and all positions with less than 85% site coverage were eliminated, resulting in 653 positions in the final dataset. The tree was drawn to scale, with branch lengths representing the number of substitutions per site (scale bar in the figure).

**
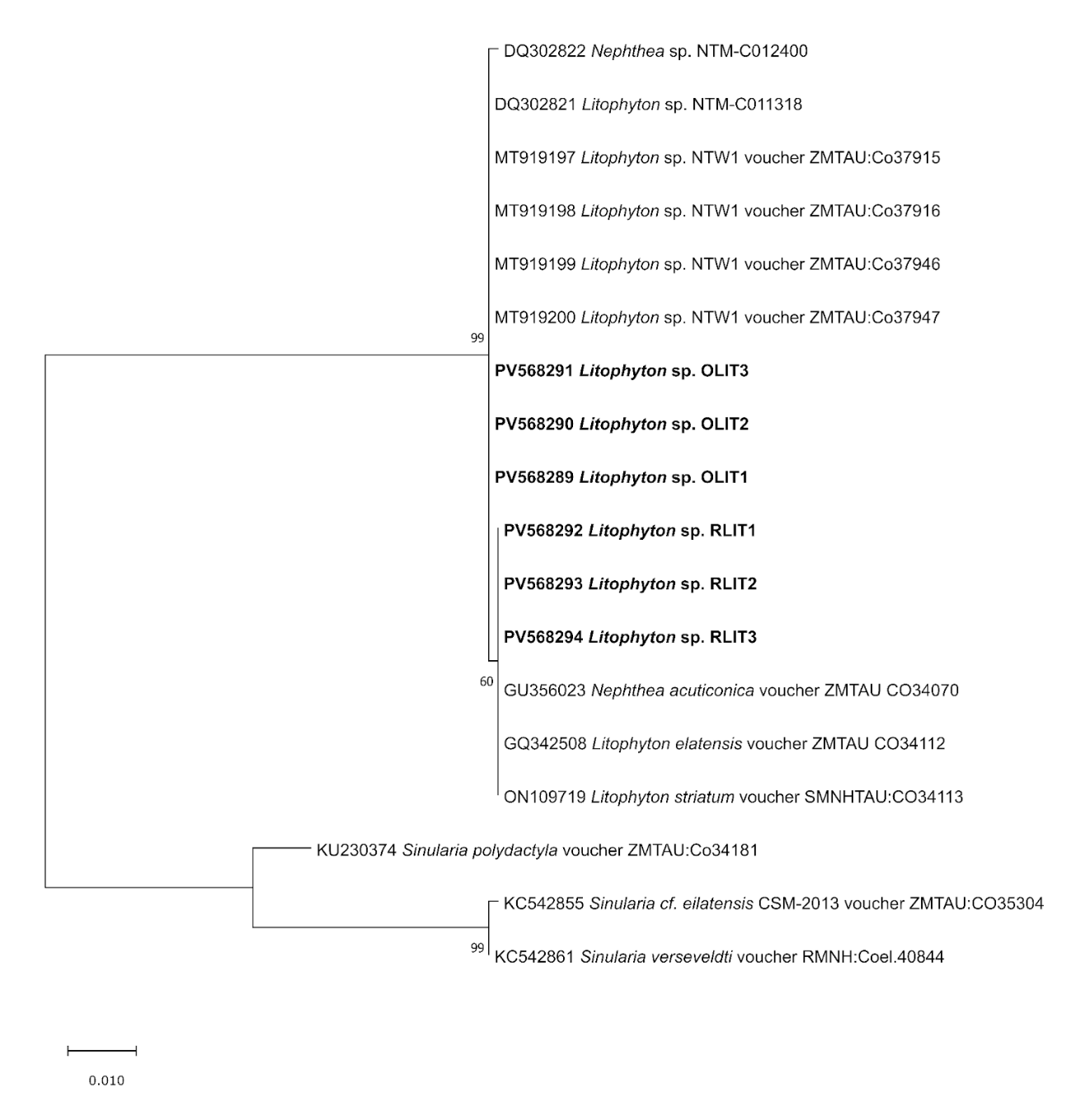
**

**Figure S3. Phylogenetic inference, based on the Mutator S (mtMutS; formerly msh1) gene, of the octocoral samples used in this study.** The tree depicts six octocoral mtMutS gene sequences from the samples here studied (OLIT1-OLIT3 from aquarium corals and RLIT1-RLIT3 from Red Sea corals, all in bold), and nine voucher mtMutS gene sequences of the genus *Litophyton* (formerly *Nephthea*). Moreover, three voucher mtMutS gene sequences of the genus *Sclerophytum* (previously *Sinularia*) were used as an outgroup to root the tree. All voucher sequences were publicly available and retrieved from Genbank, NCBI. The tree was constructed using the Maximum-Likelihood algorithm with the Tamura 3-parameter model, with uniform rates and 1,000 bootstrap repetitions. Codon positions included were 1st+2nd+3rd+Noncoding. Partial deletion was used and all positions with less than 85% site coverage were eliminated, resulting in 735 positions in the final dataset. The tree was drawn to scale, with branch lengths representing the number of substitutions per site (scale bar in the figure).


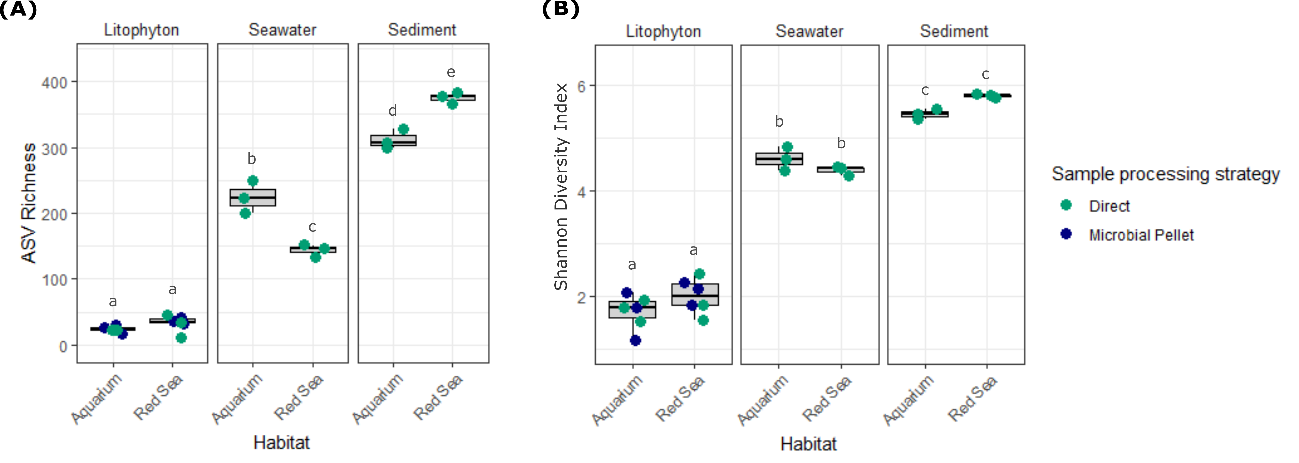


**Figure S4. Alpha-diversity analysis of rarefied prokaryotic communities associated with Litophyton sp. and the surrounding environment, including sample RLIT1.** (A) ASV richness and (B) Shannon diversity indices are compared across biotopes using the rarefied dataset with a threshold of 466 sequences. The coloring of the dots indicates the sampling processing strategy: green – direct DNA extraction from holobiont sample, blue – DNA extracted from microbial pellets. Different letters above box plots indicate significant differences between habitats for each biotope and index (Two-Way ANOVA followed by a Tukey’s HSD post-hoc test if significant; p < 0.05).

**
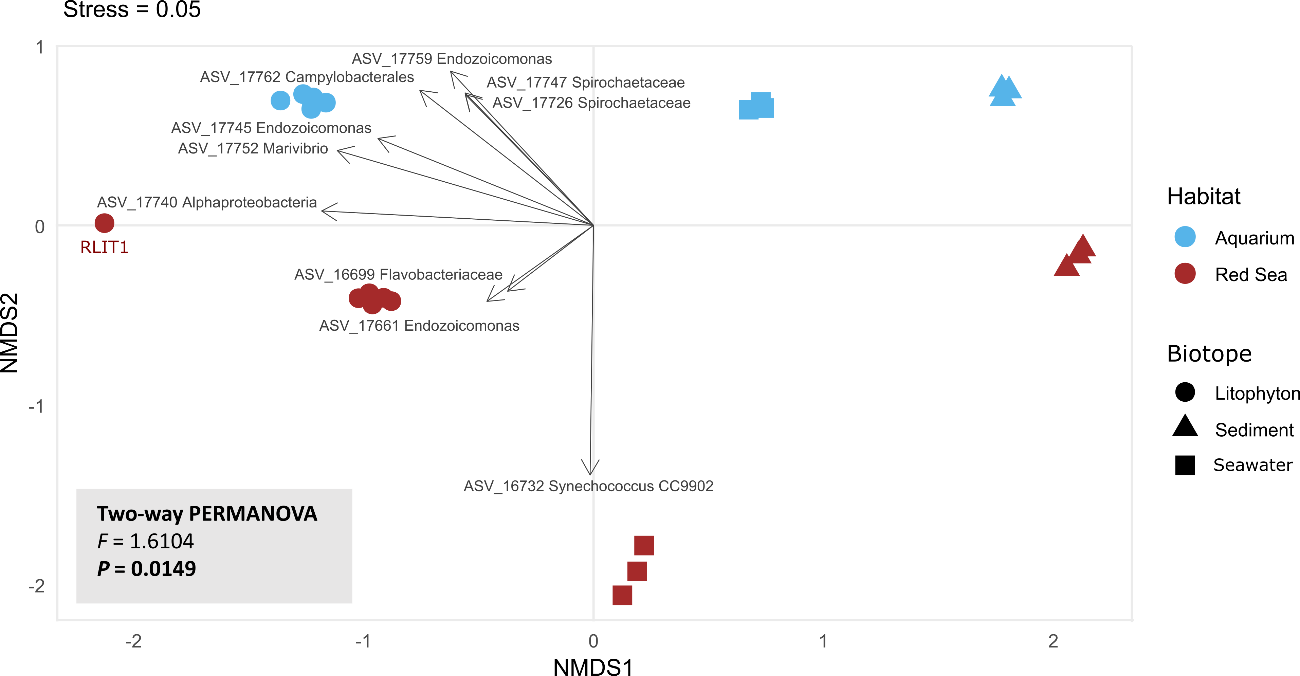
**

**Figure S5. Multivariate analysis of the rarefied prokaryotic community profiles, including sample RLIT1.** Non-Metric Multidimensional Scaling (NMDS) ordination plot at the ASV-level. The plot shows the ordination based on a Bray-Curtis similarity matrix calculated from (non-rarefied) Hellinger-transformed abundance data. Aquarium and Red Sea samples are represented by blue and red symbols, respectively. Biotopes are indicated by different shapes: circles for Litophyton (RLIT1-3, MPRLIT1-3, OLIT1-3, MPOLIT1-3), squares for seawater (OASW1-3, RSW1-3), and triangles for sediments (OSED1-3, RSED1-3). Black arrows represent the top ten ASVs contributing most to community dissimilarities, as identified by SIMPER analysis. Differences in sample group composition were assessed using an unrestricted full-factorial PERMANOVA with 9,999 permutations to evaluate the effects of habitat, biotope, and their interaction (p<0.05), followed by pairwise PERMANOVA analyses to investigate specific group-level differences. No statistical significant differences between prokaryotic communities of the same biotope across habitats were identified.


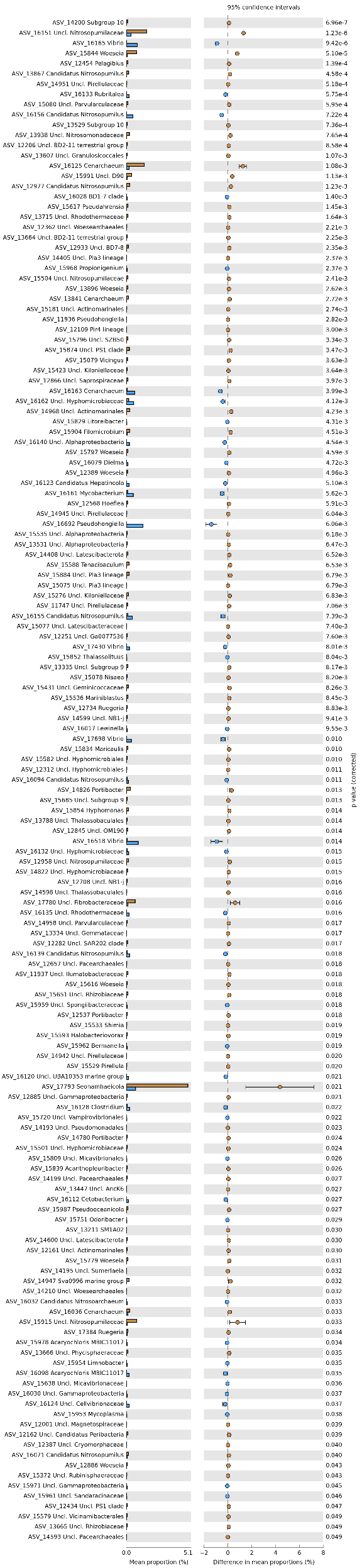
**Figure S6. Differentially abundant prokaryotic ASVs among aquarium biotopes.** Panels depict pairwise comparisons of prokaryotic non-rarefied ASVs differentially abundant between biotopes: A) Litophyton vs sediments, B) Litophyton vs artificial seawater, and C) sediments vs seawater. Biotopes are represented by distinct colors: Sediments - brown, Litophyton – pink, and seawater - blue. Significant ASVs were identified using a two-sided Welch’s t-test (p-value < 0.05), with an additional effect size filter applied by setting the 'ratio of proportions' threshold to 2.0.


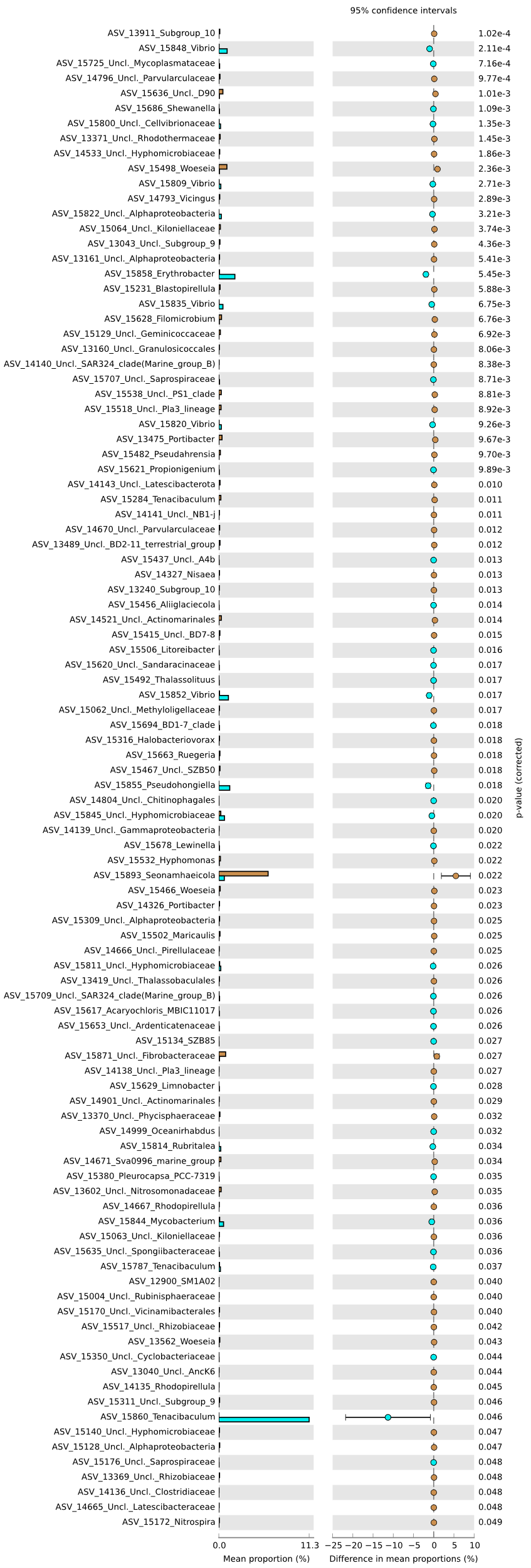

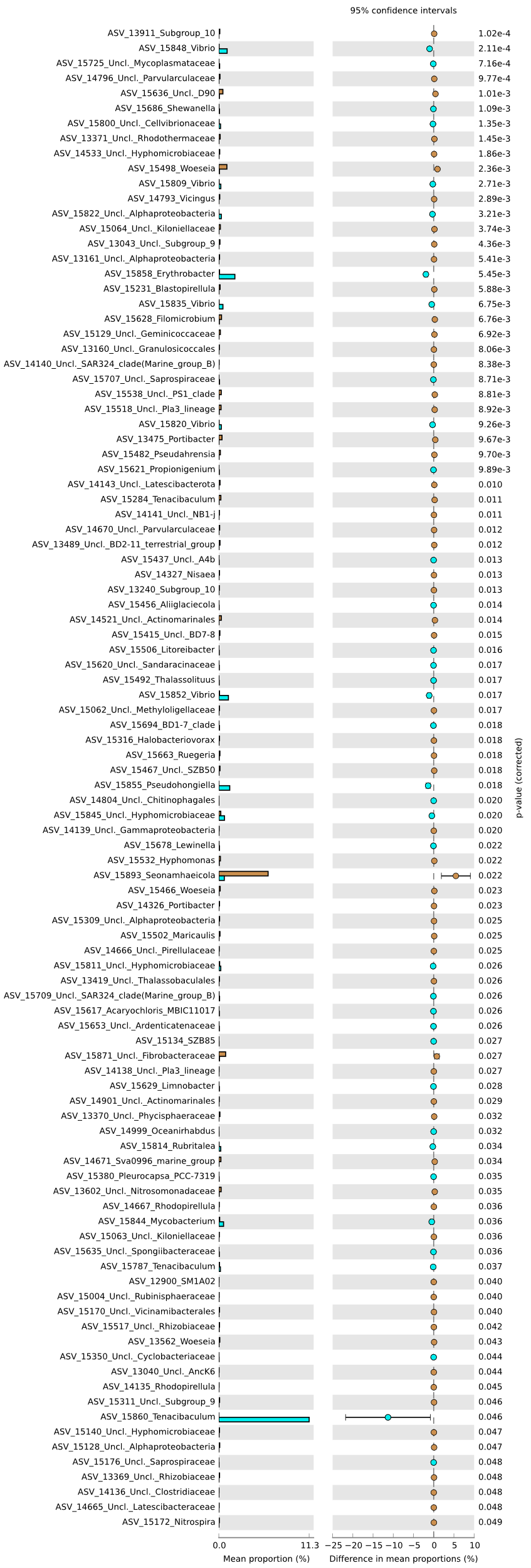

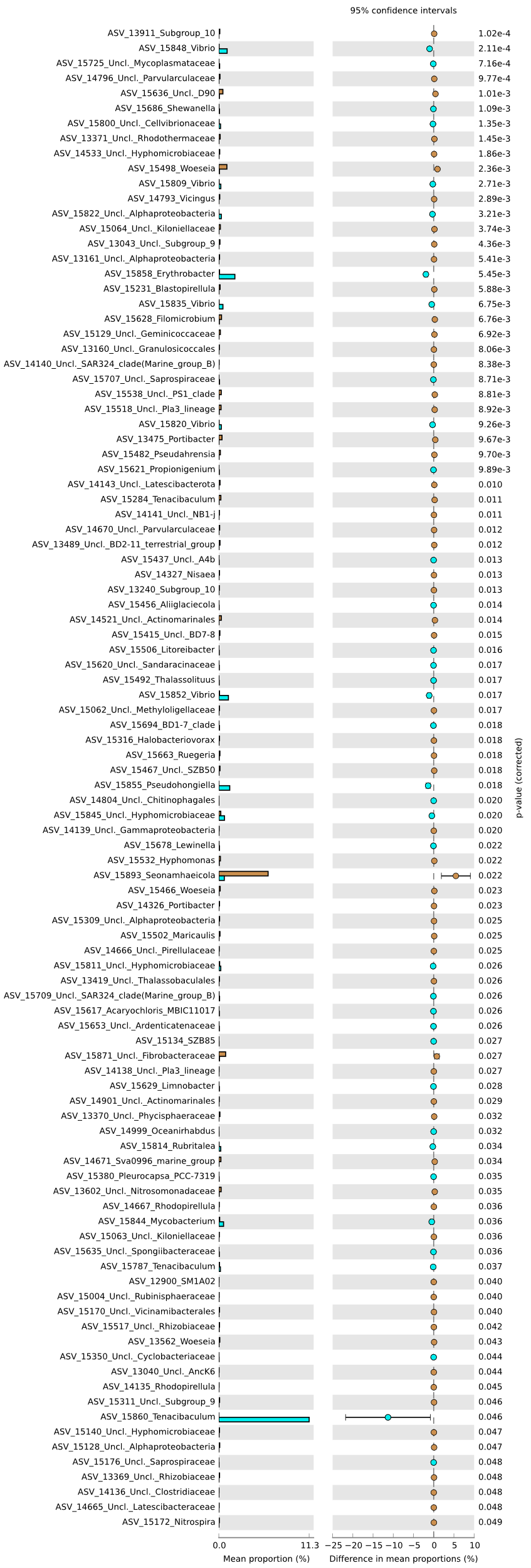


**(A)**

**(B)**

**(C)**

Sediment

*Litophyton*

Seawater


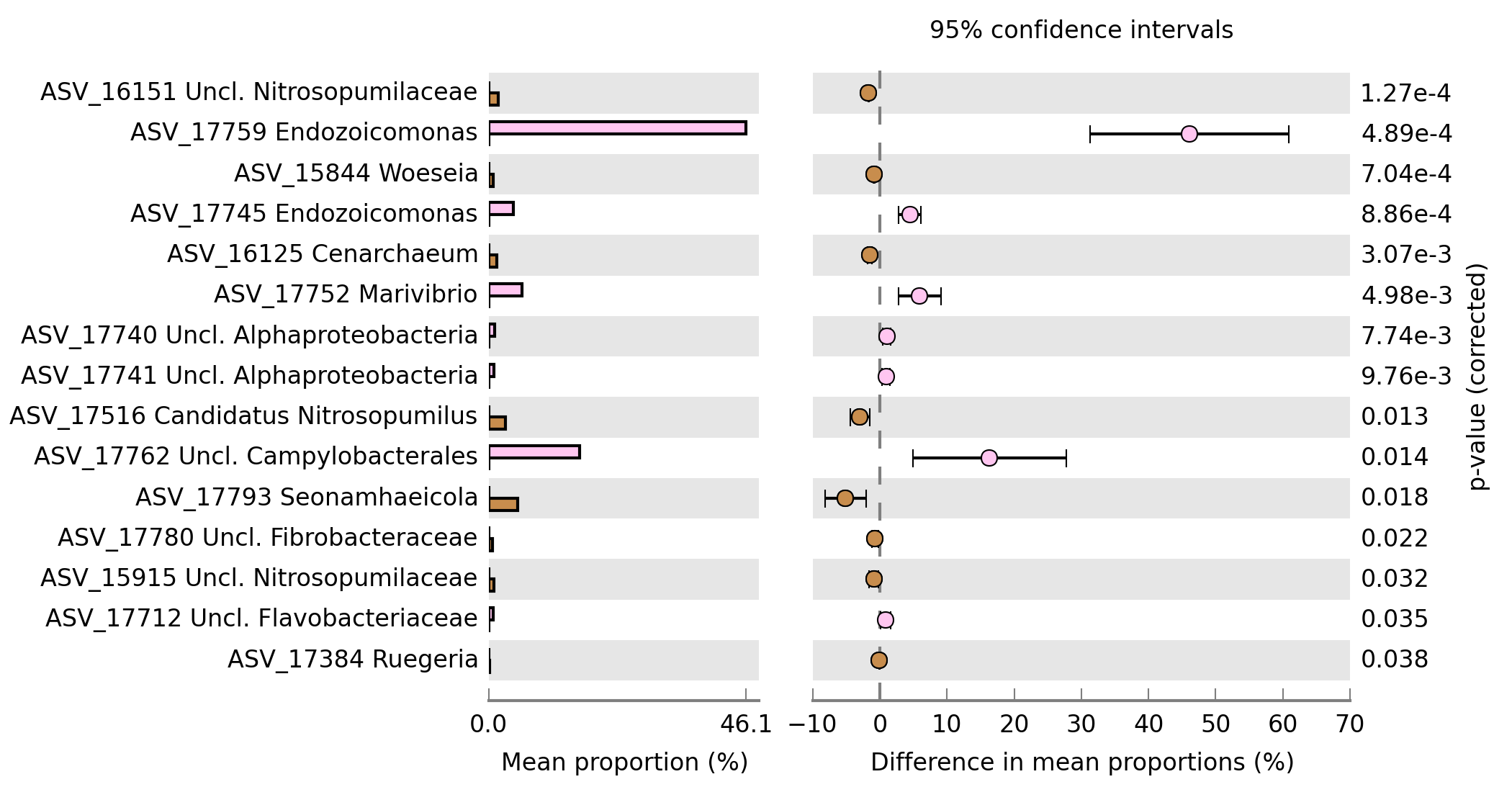

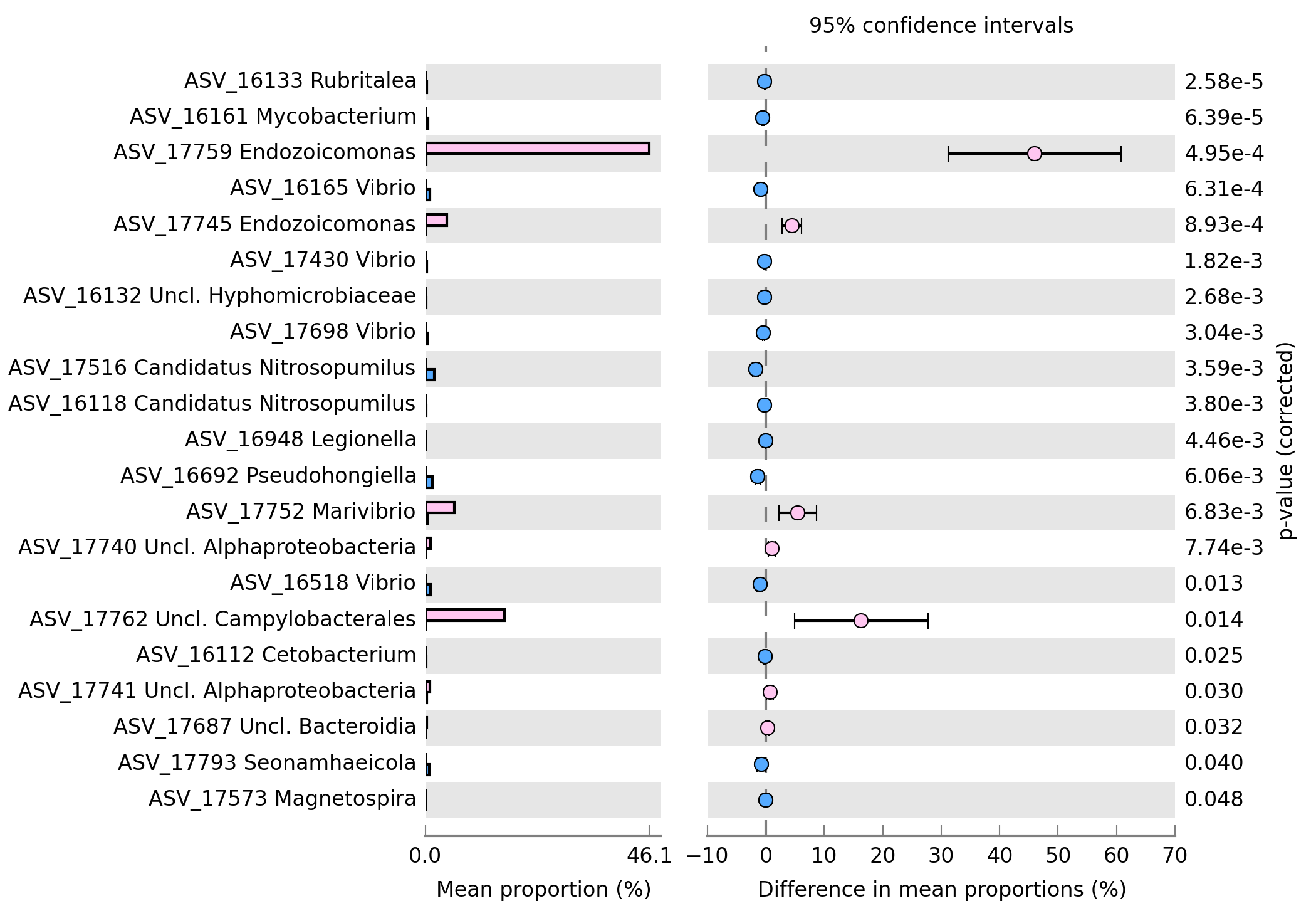

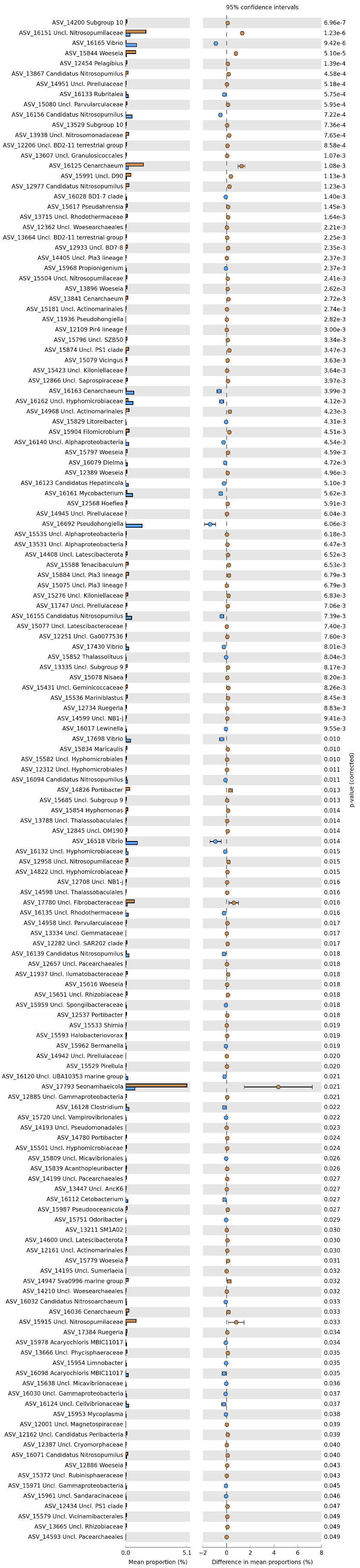

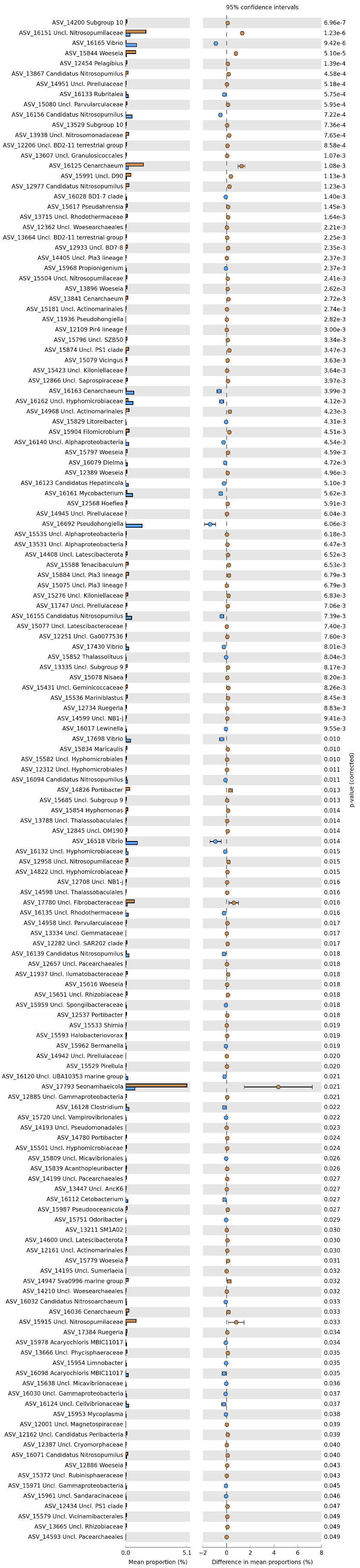

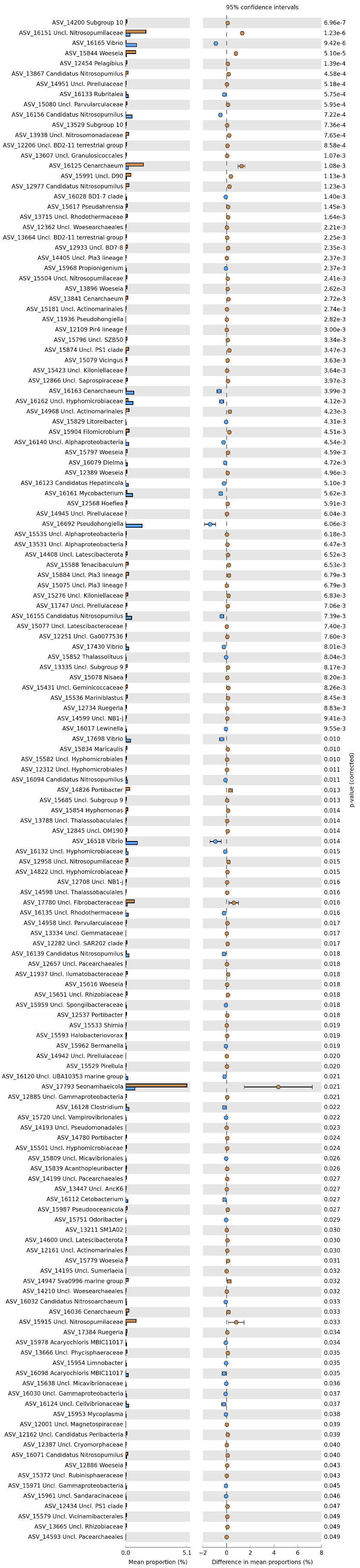

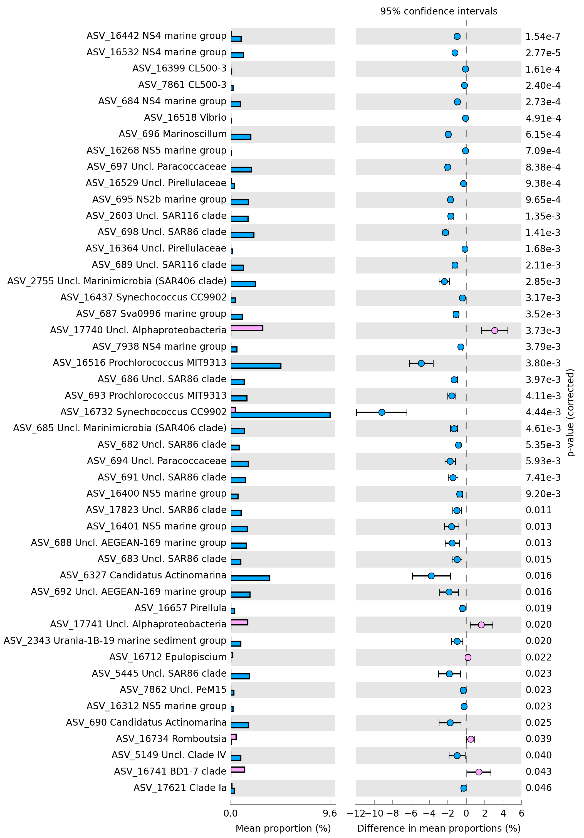

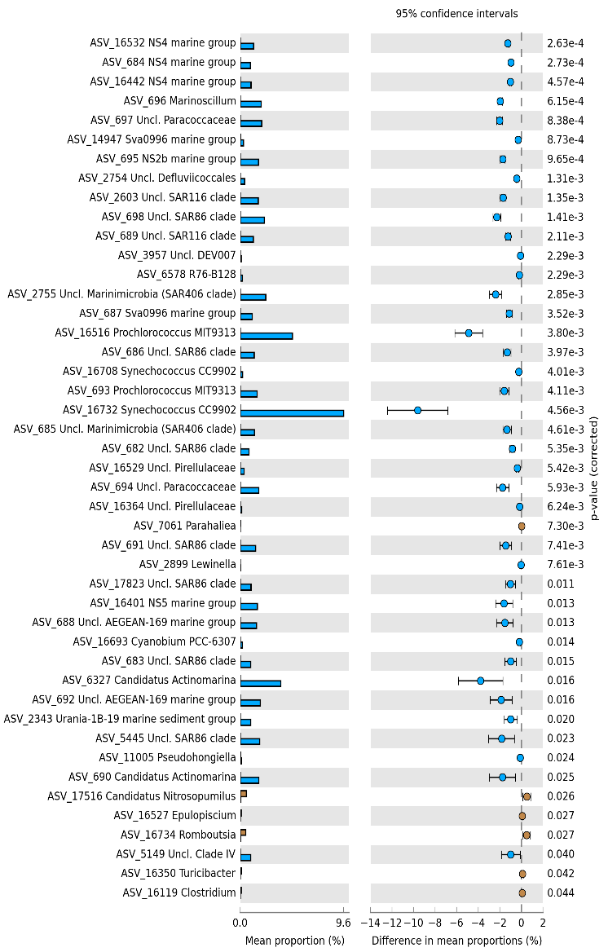


**(A)**

**(B)**

**(C)**

Sediment

*Litophyton*

Seawater


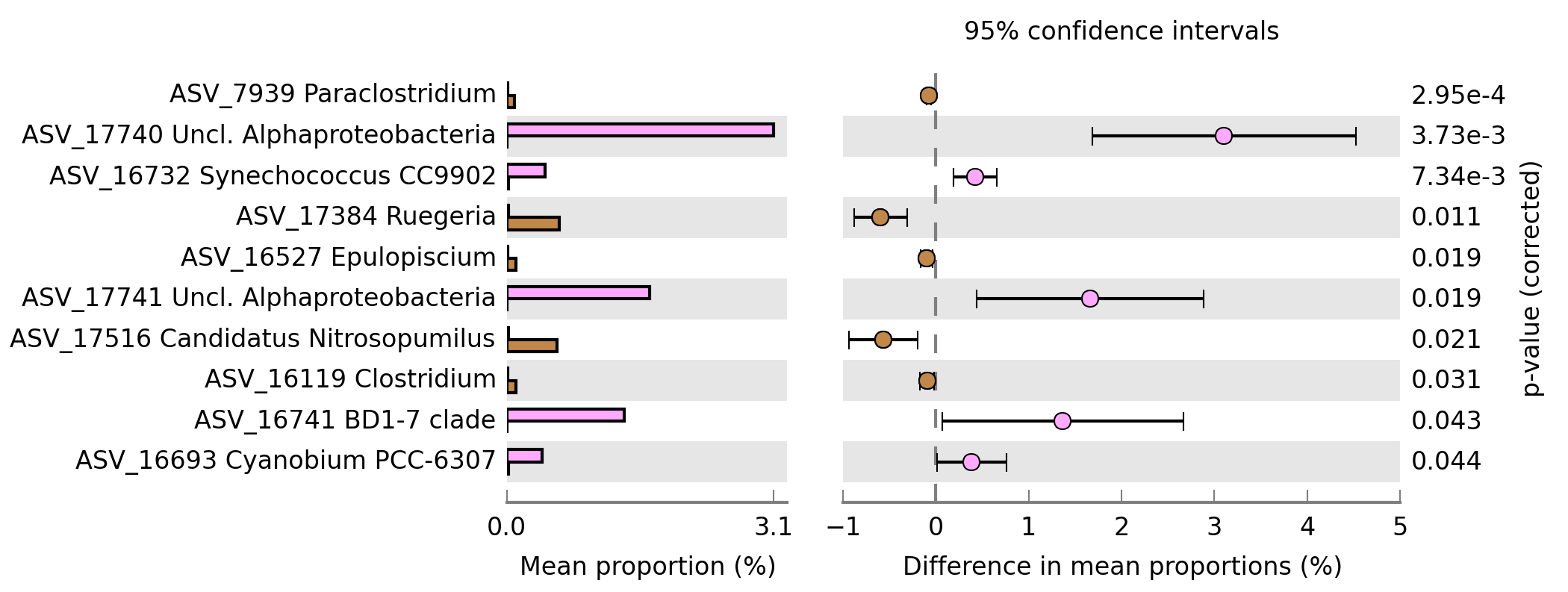


**Figure S7. Differentially abundant prokaryotic ASVs among Red Sea biotopes, excluding sample RLIT1.** Panels depict pairwise comparisons of prokaryotic non-rarefied ASVs that were found to be differentially abundant between biotopes: A) Litophyton vs sediments, B) Litophyton vs seawater, and C) sediments vs seawater. Biotopes are represented by distinct colors: Sediments - brown, Litophyton – pink, and seawater - blue. Significant ASVs were identified using a two-sided Welch’s t-test (p-value < 0.05), with an additional effect size filter applied by setting the 'ratio of proportions' threshold to 2.0.


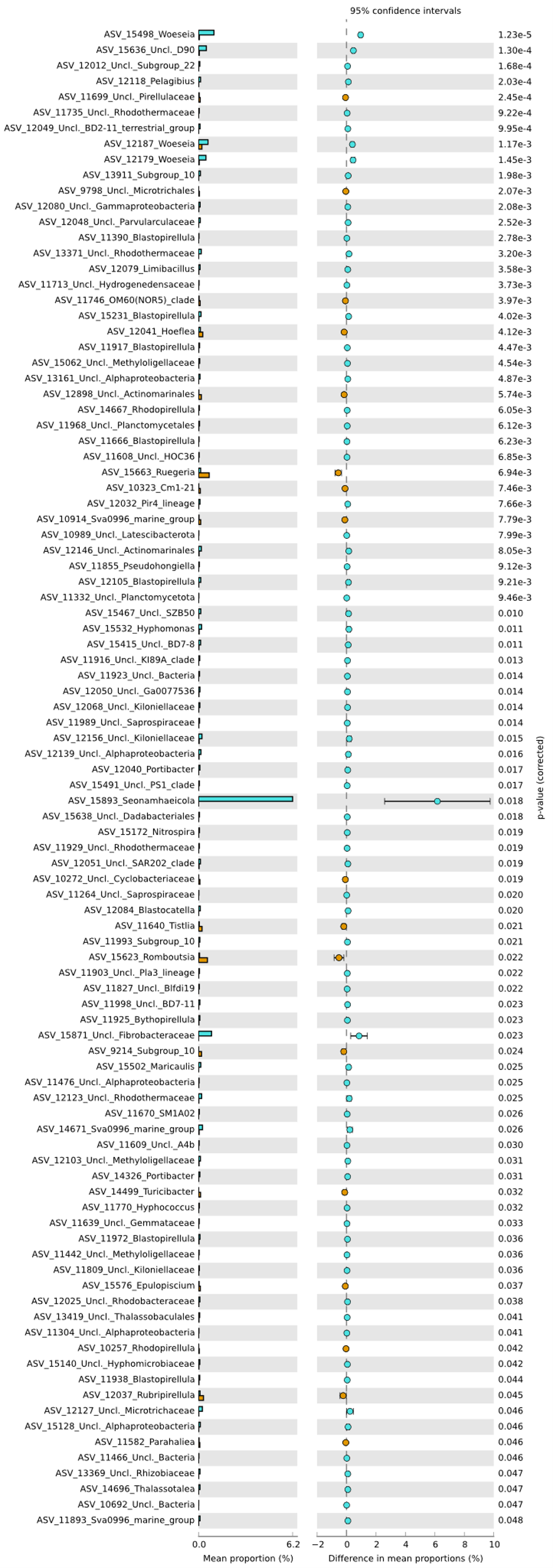

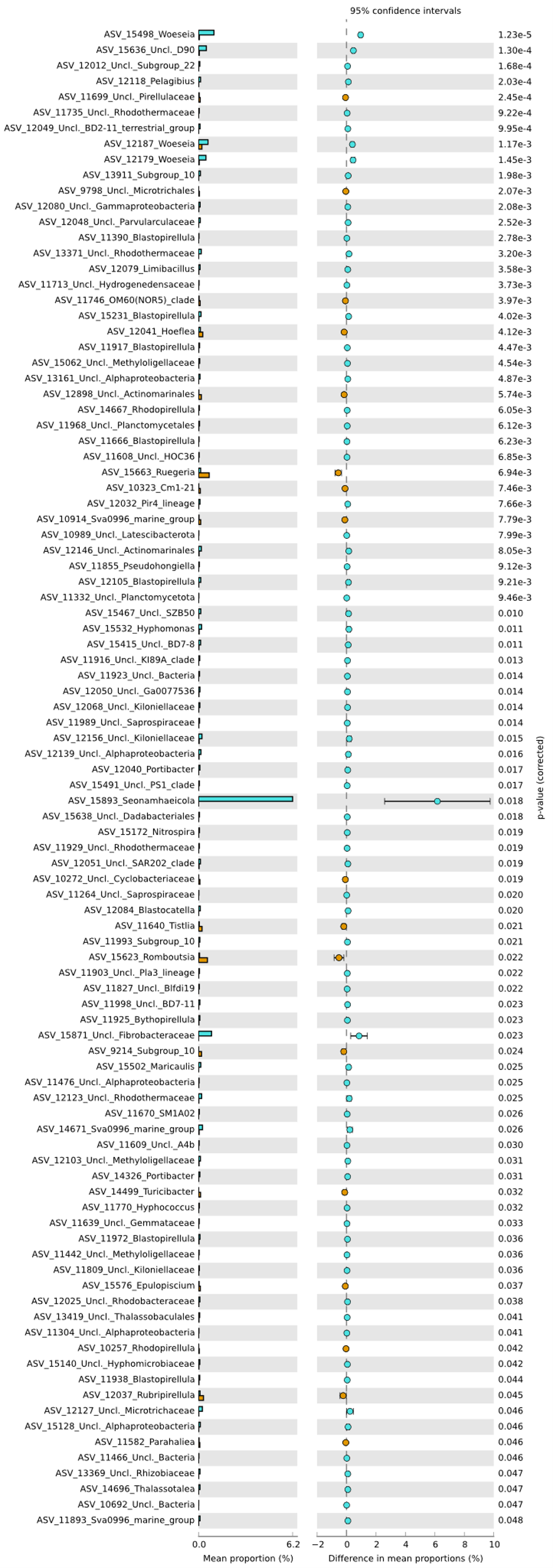


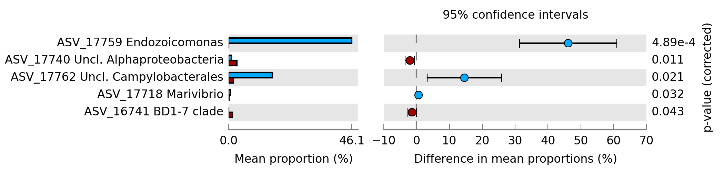

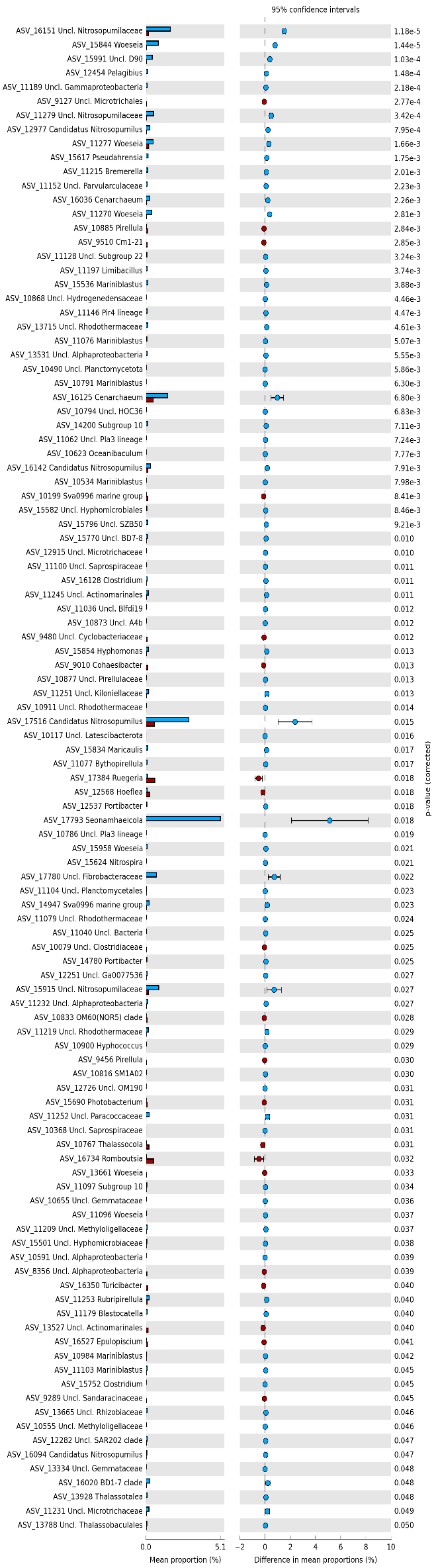

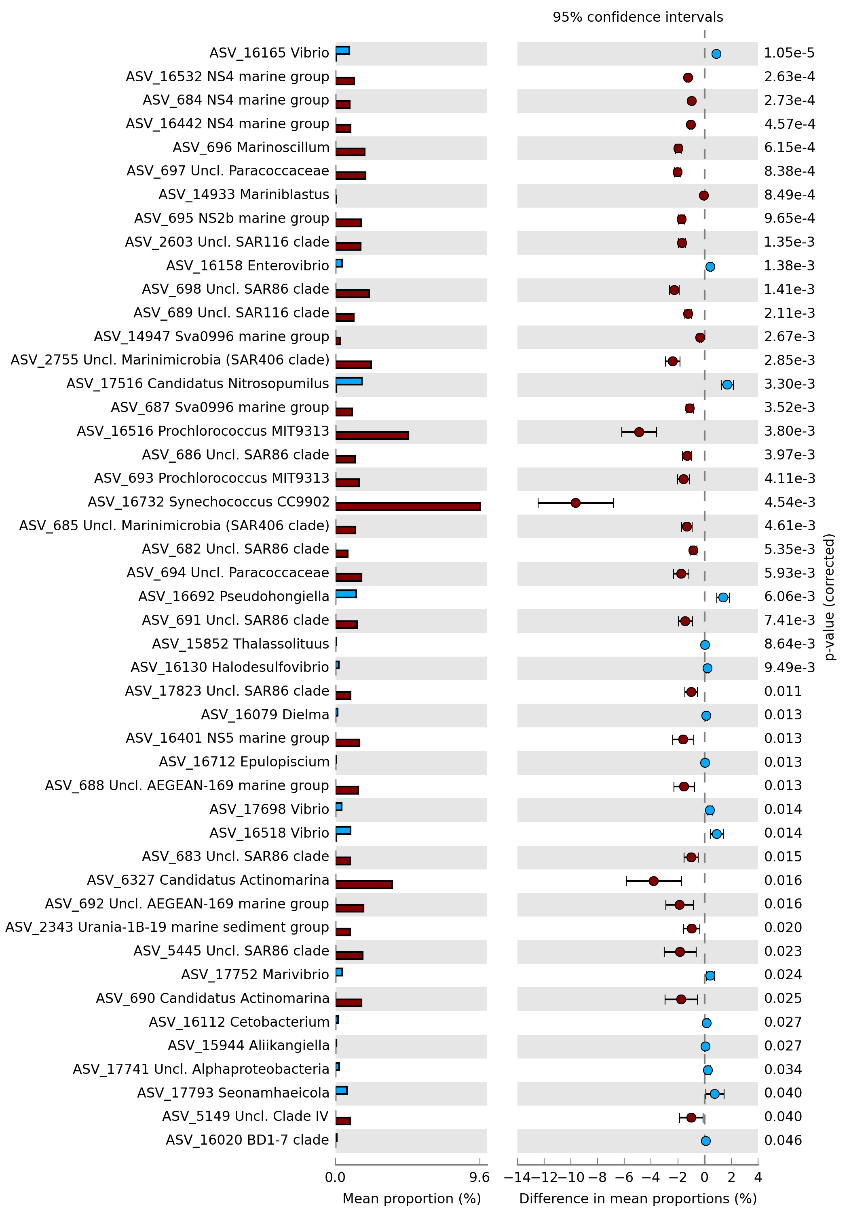

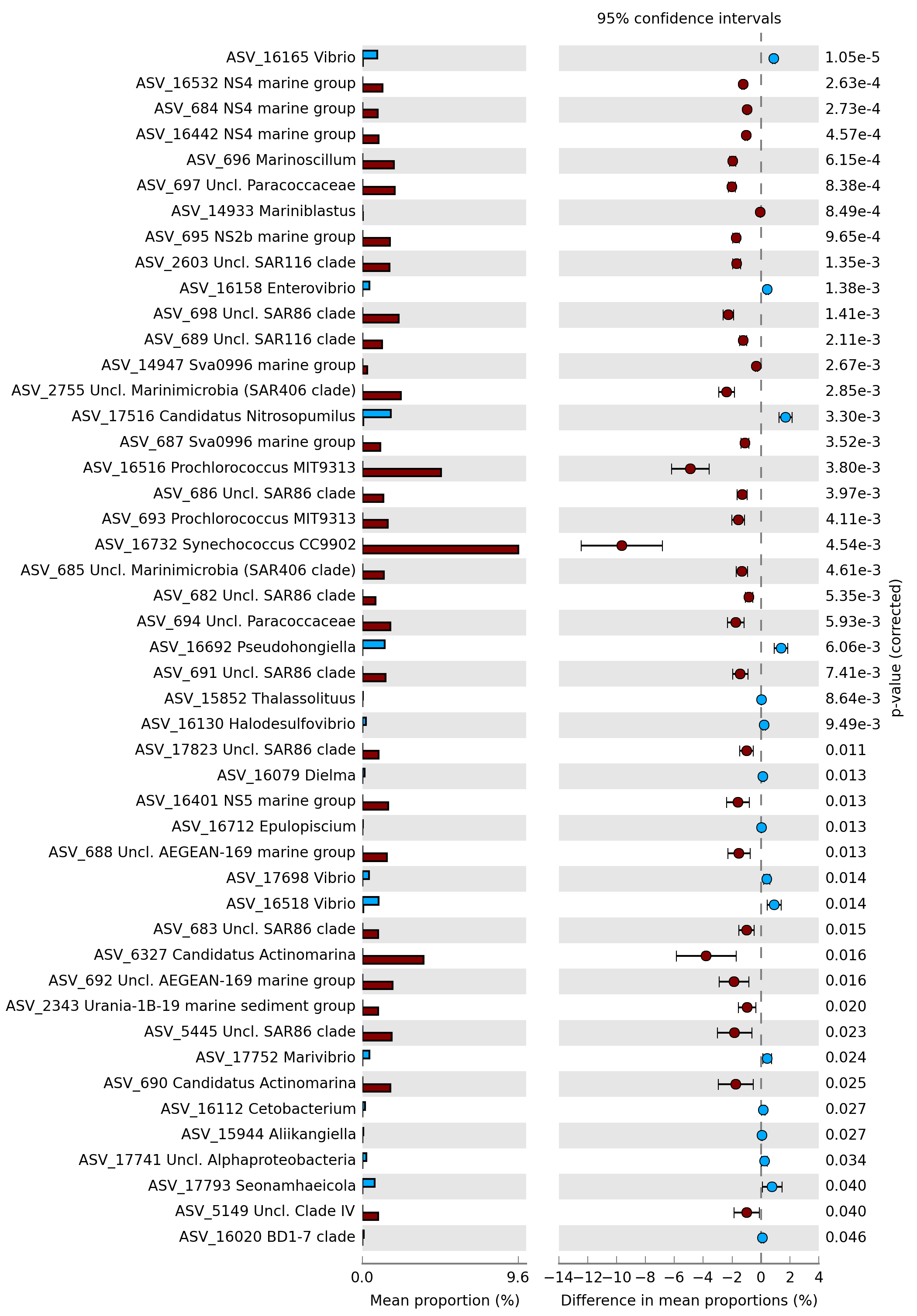

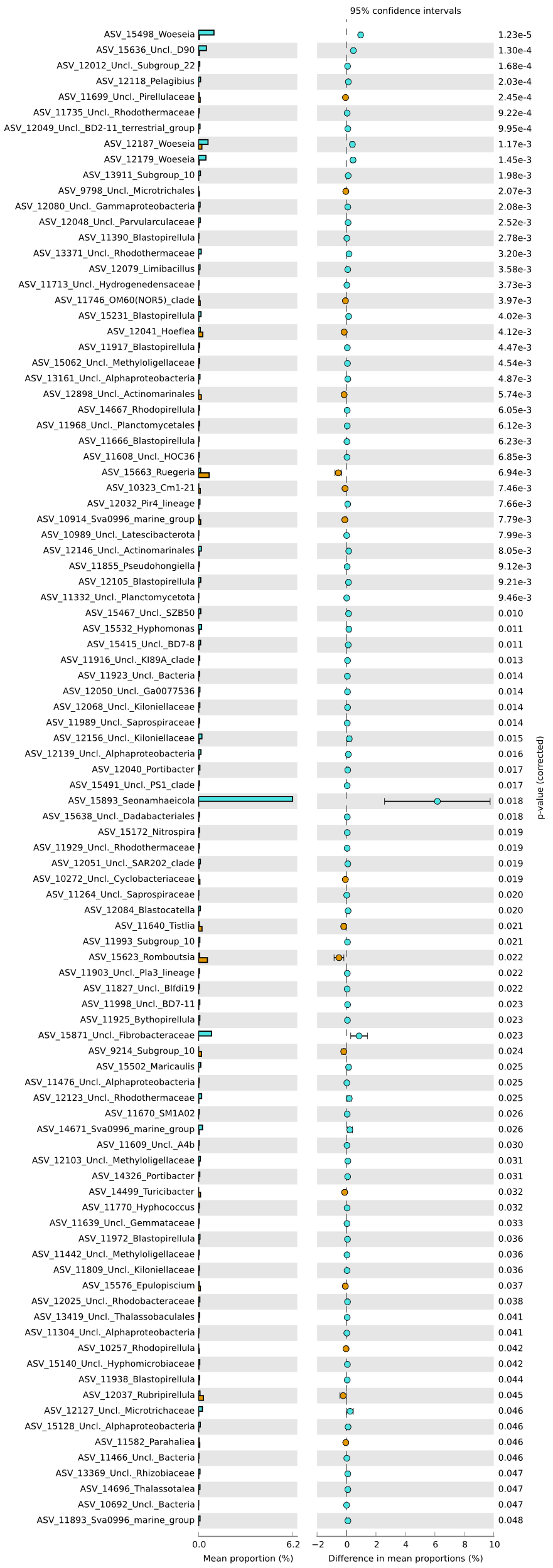

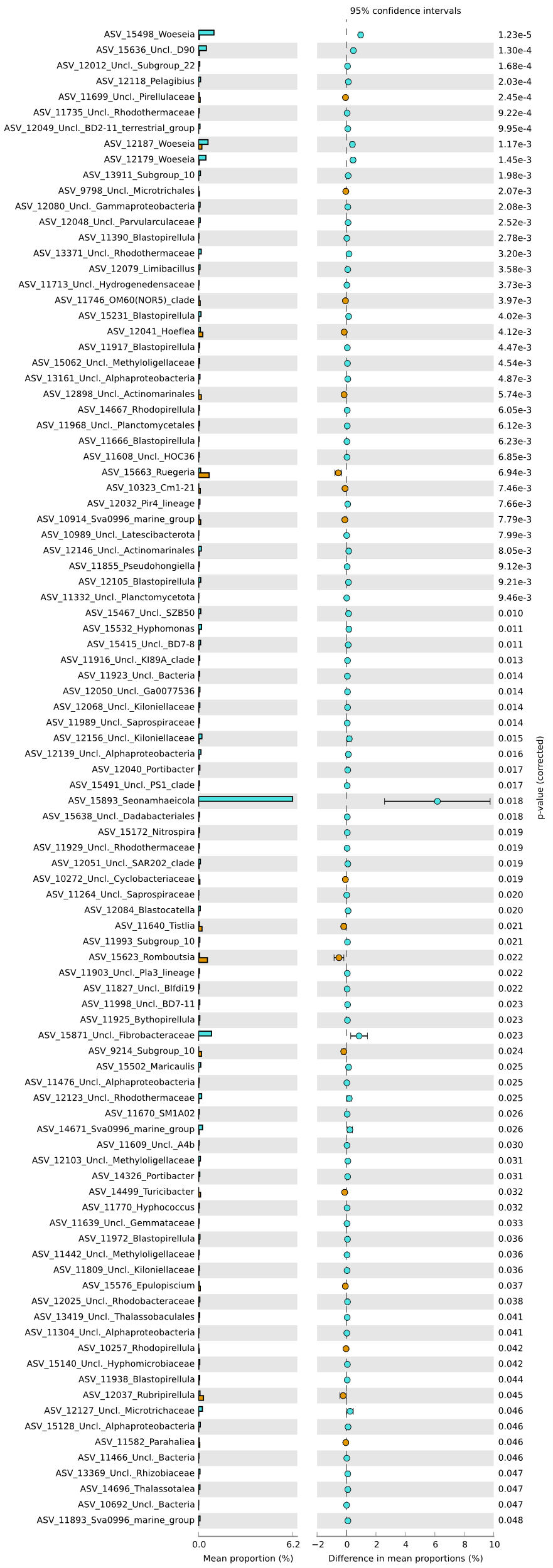

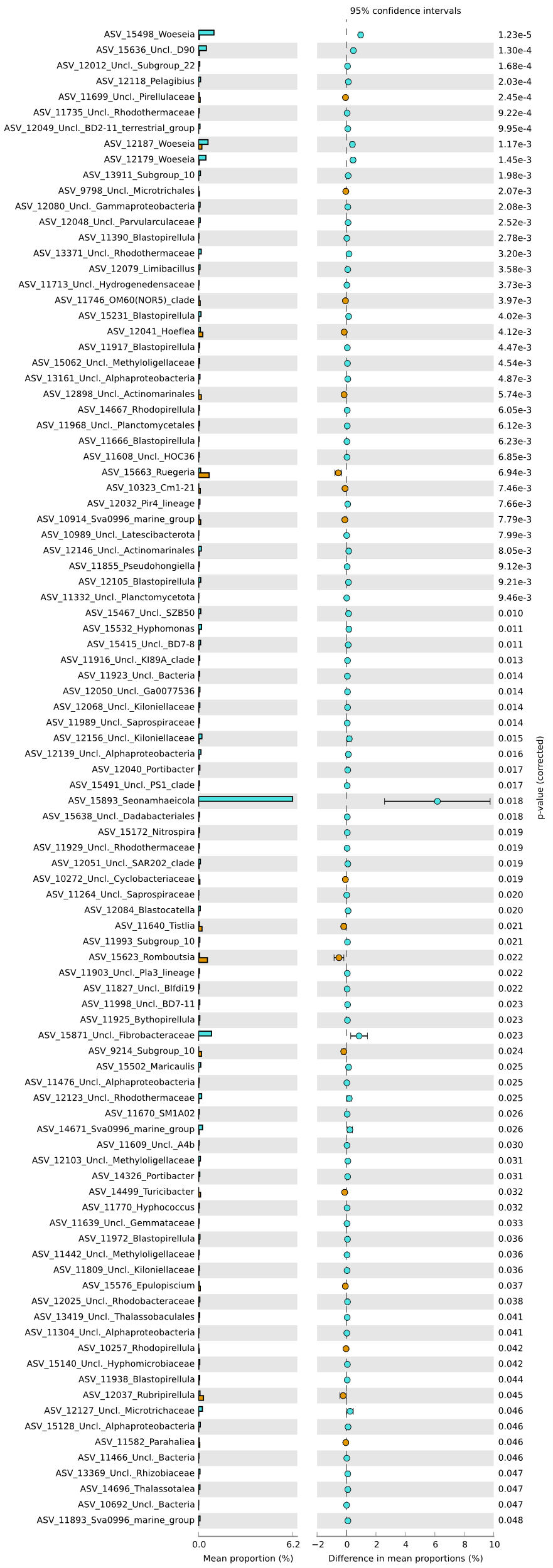

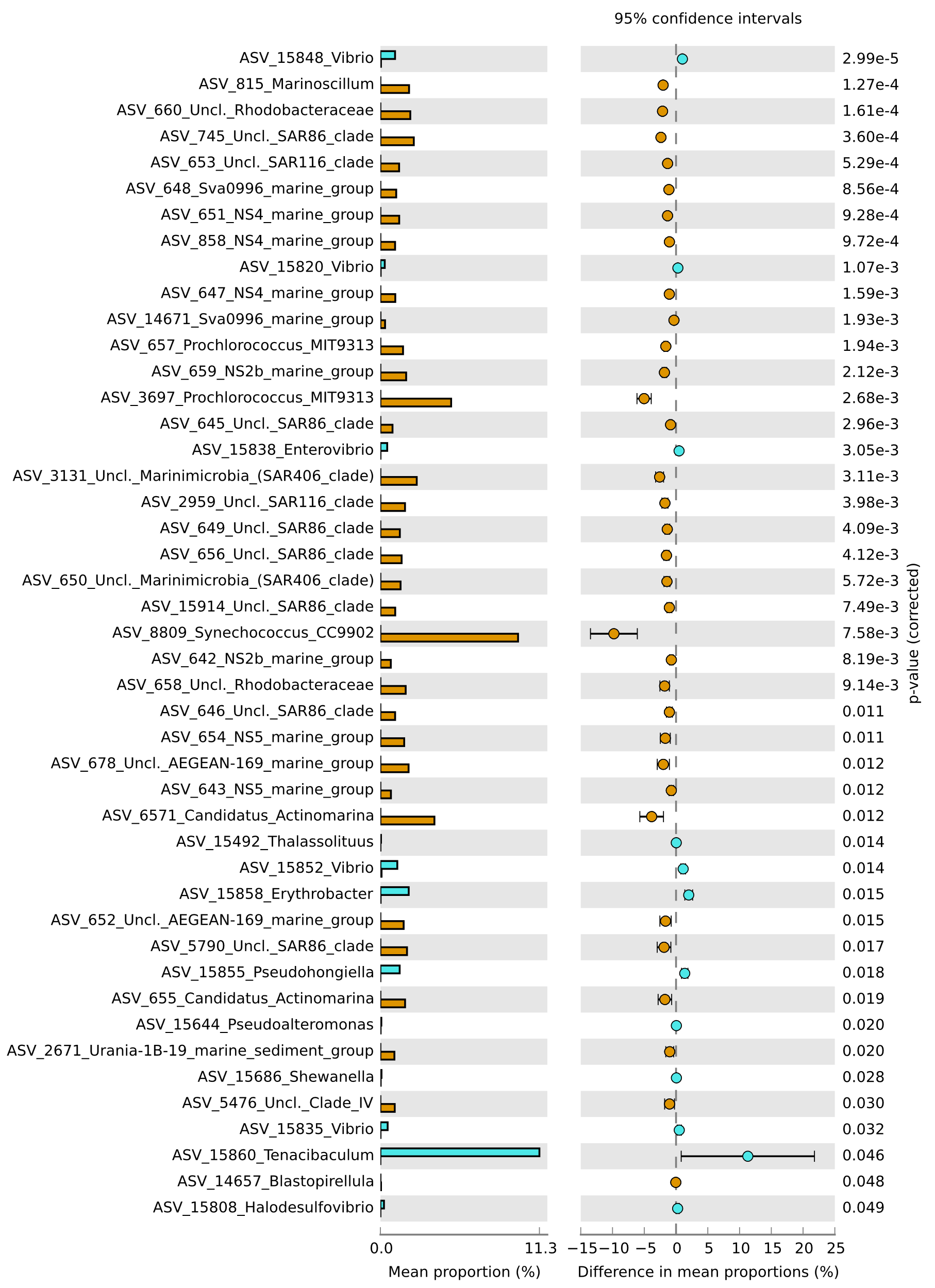

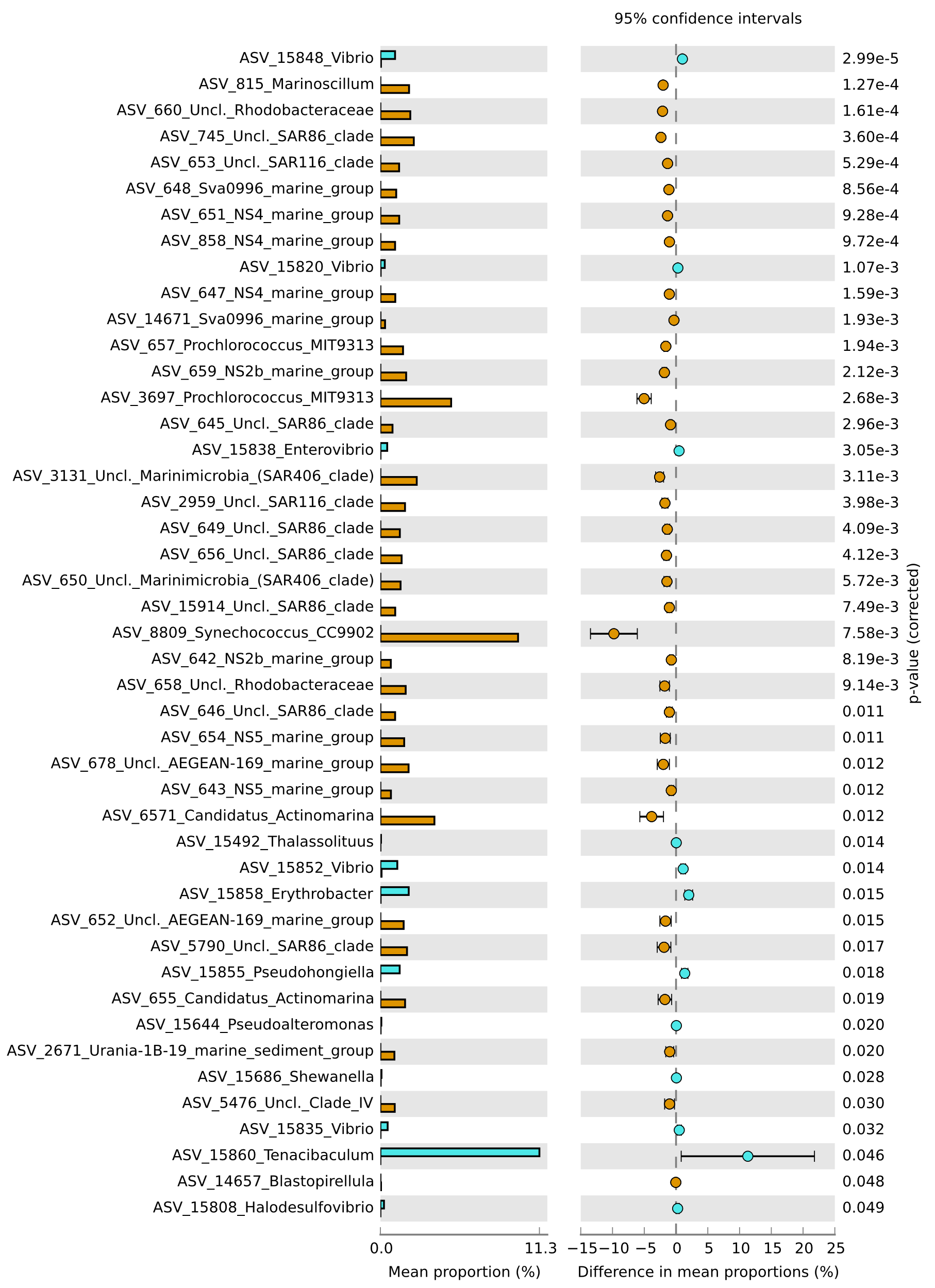


Red Sea

Aquarium

**(B)**

**(C)**

**(A)**


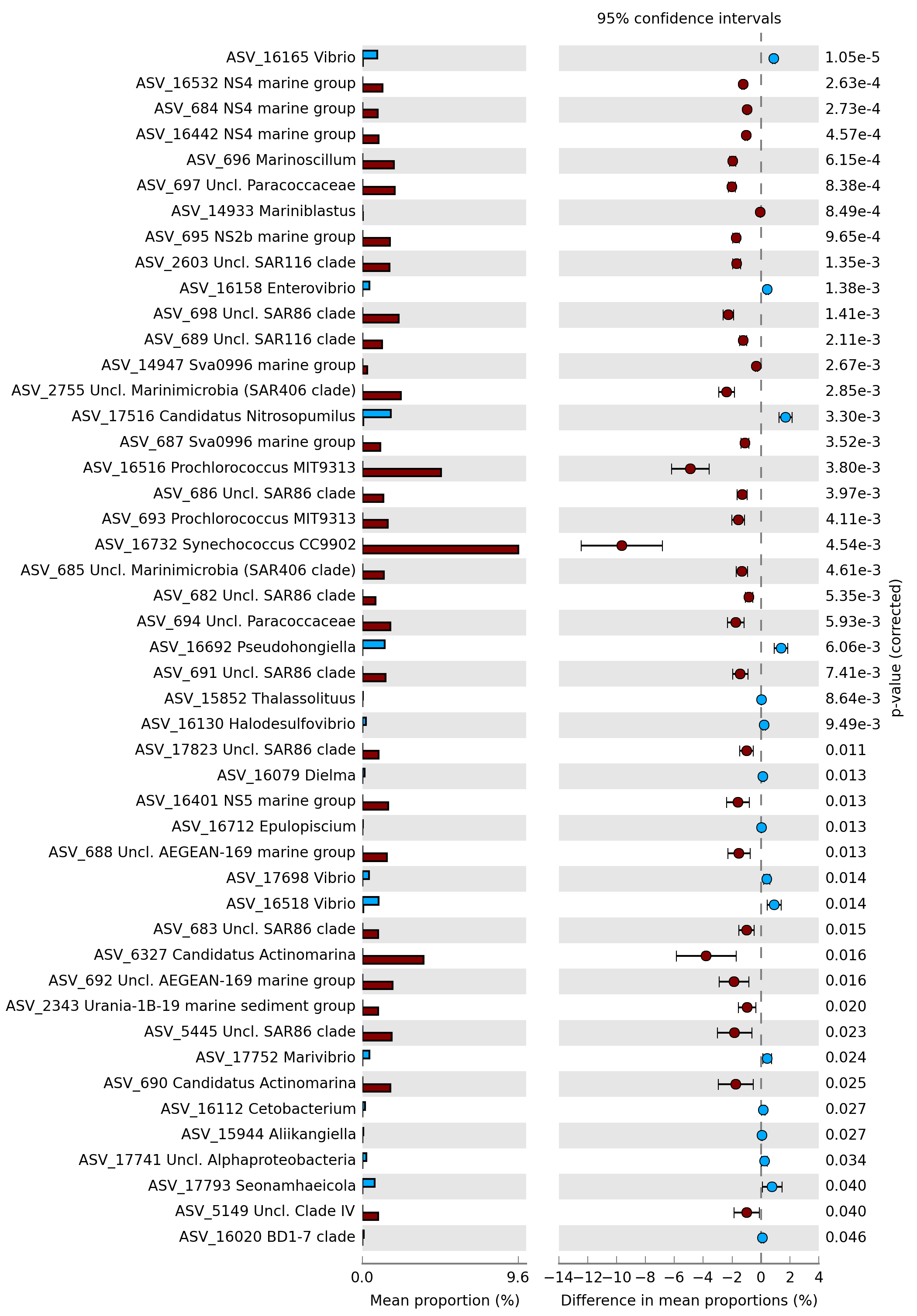

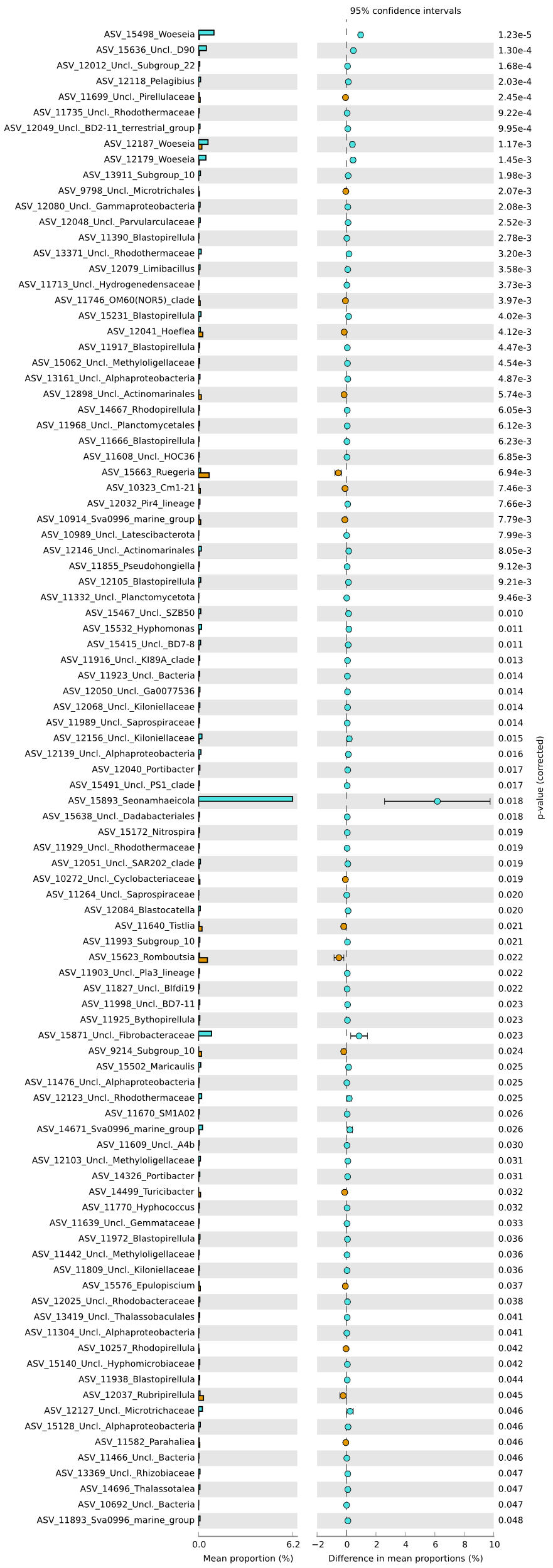

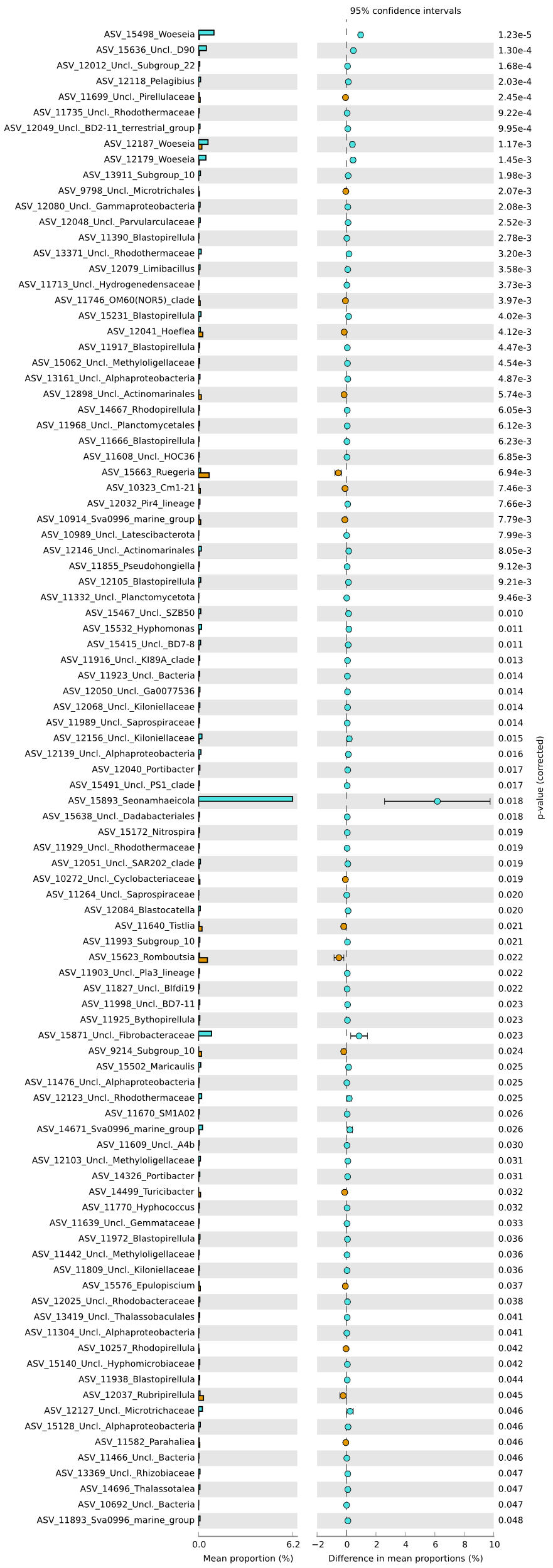

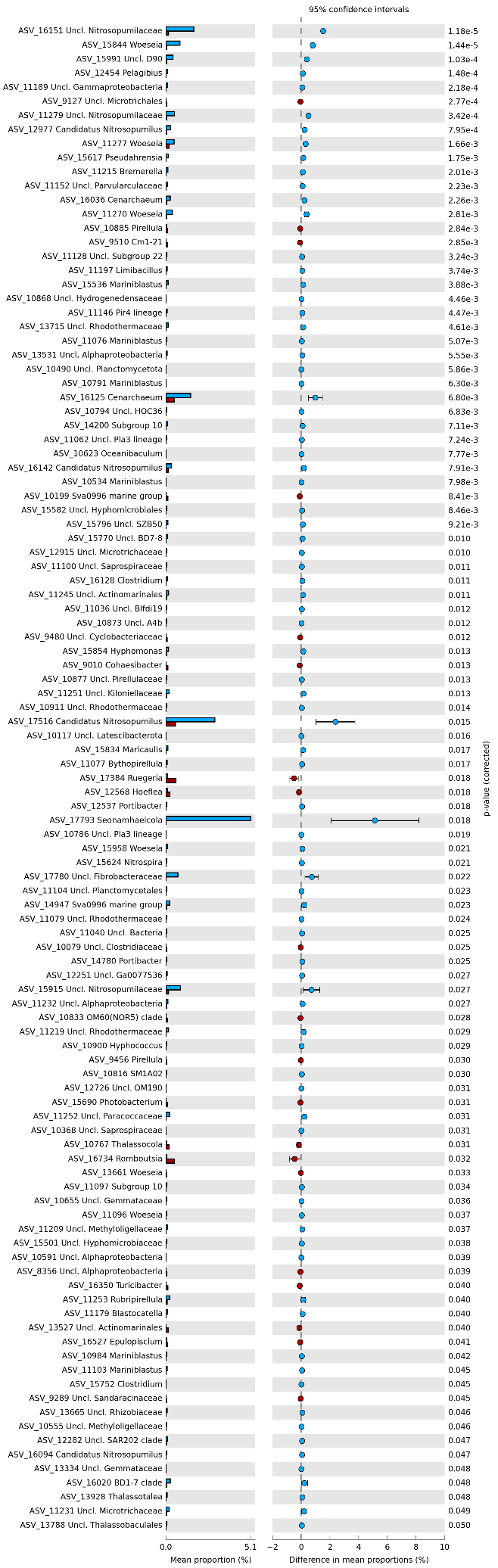

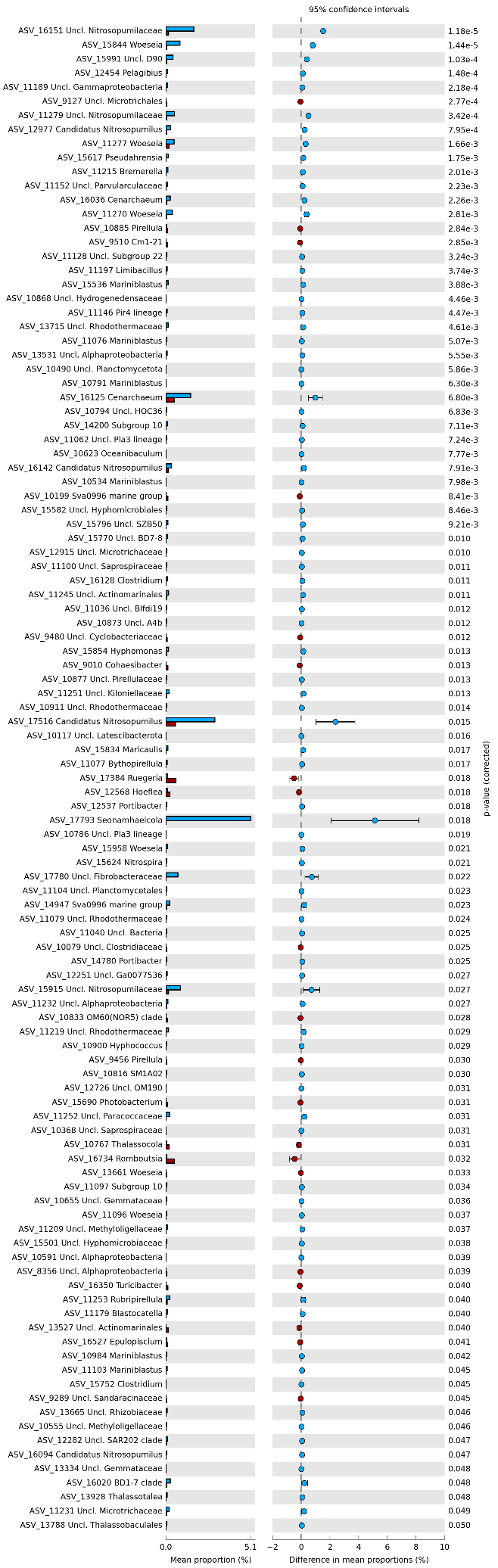


**Figure S8. Differentially abundant prokaryotic ASVs of each biotope across habitats.** Panels depict pairwise comparisons of prokaryotic non-rarefied ASVs differentially abundant between habitats for each biotope: A) sediments, B) seawater, and C) Litophyton (sample RLIT1 not included in analysis). Habitats are represented by distinct colors: Red Sea - red, Aquarium – blue. Significant ASVs were identified using a two-sided Welch’s t-test (p-value < 0.05), with an additional effect size filter applied by setting the 'ratio of proportions' threshold to 2.0.


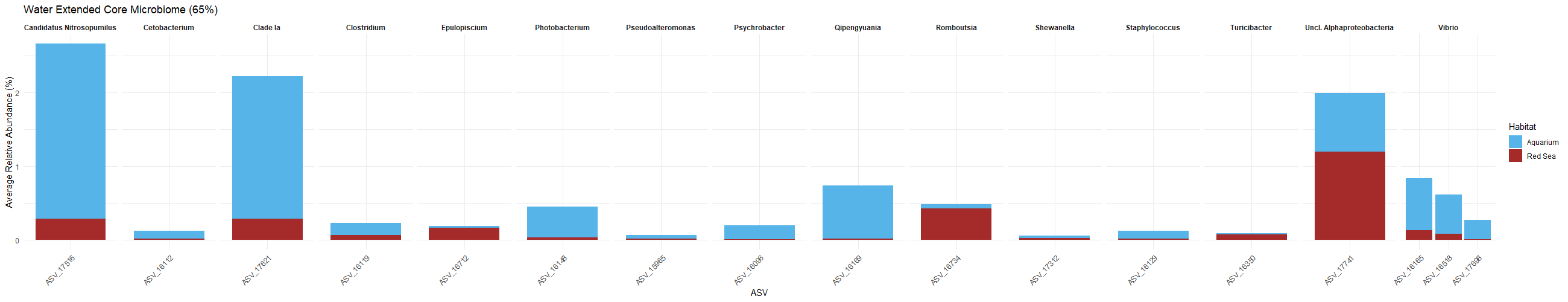


**(B)**


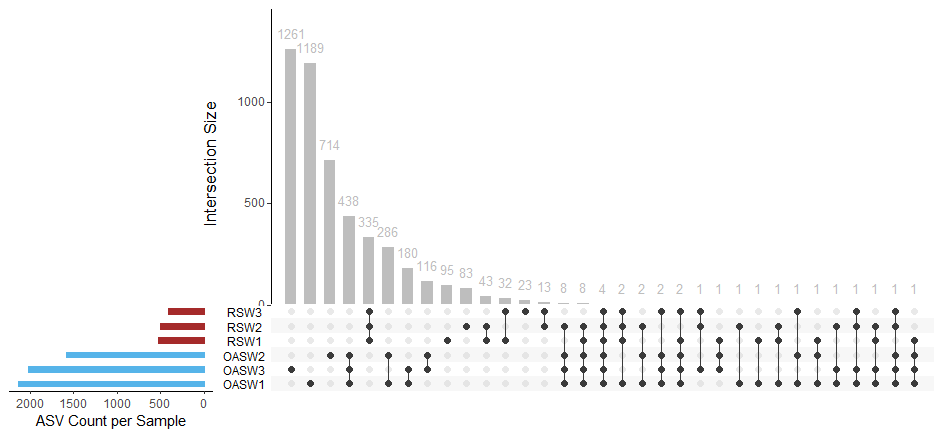


**(A)**

**Figure S9. Core prokaryotic ASVs in seawater samples.** Aquarium samples are represented in blue, while Red Sea samples are shown in red. A) Intersection plot illustrating the number of ASVs unique to individual samples (single dots) and shared across multiple samples (connected dots). B) Bar chart displaying the relative abundances of ASVs present in at least 65% seawater samples per habitat.


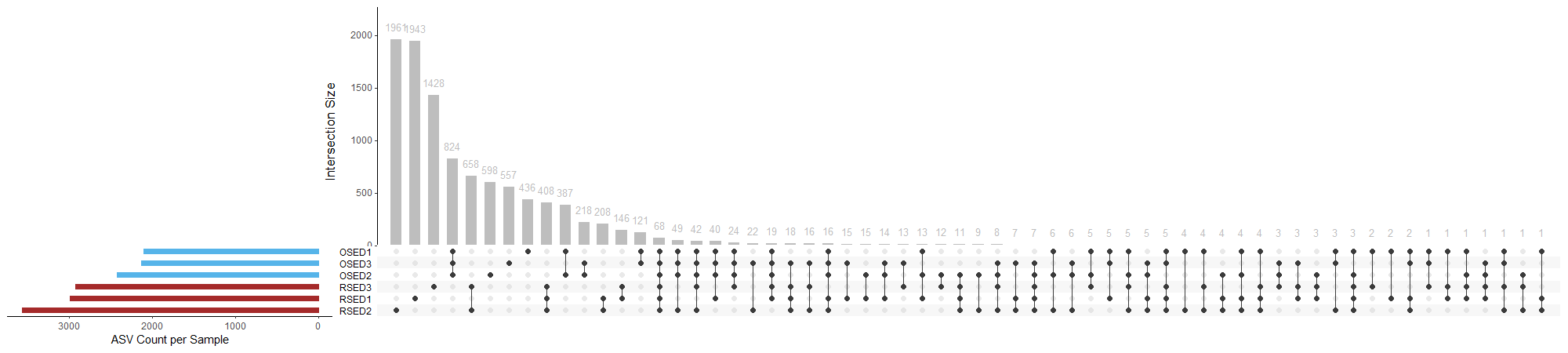


**(A)**

**(B)**


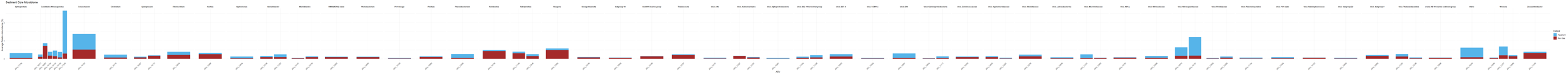

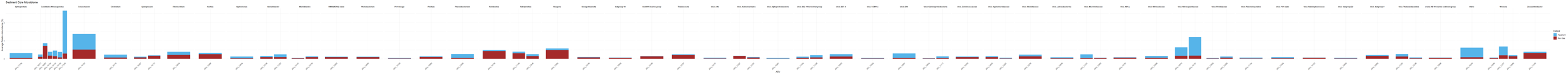


**Figure S10. Core prokaryotic ASVs in sediment samples.** Aquarium samples are represented in blue, while Red Sea samples are shown in red. A) Intersection plot illustrating the number of ASVs unique to individual samples (single dots) and shared across multiple samples (connected dots). B) Bar chart displaying the relative abundances of ASVs present in 100% of sediment samples.

**References**

Esteves, A.I.S., Hardoim, C.C.P., Xavier, J.R., Gonçalves, J.M.S., Costa, R., (2013). Molecular richness and biotechnological potential of bacteria cultured from *Irciniidae* sponges in the north-east Atlantic. FEMS Microbiol Ecol 85, 519–536. doi:10.1111/1574-6941.12140

France, S.C., Hoover, L.L., (2002). DNA sequences of the mitochondrial COI gene have low levels of divergence among deep-sea octocorals (Cnidaria: Anthozoa). Hydrobiologia 471, 149–155. doi: 10.1023/A:1016517724749

Hall, T.A., (1999). BioEdit: A user-friendly biological sequence alignment editor and analysis program for Windows 95/98/NT. Nucleic Acids Symp Ser 41, 95–98.

Kumar, S., Stecher, G., Li, M., Knyaz, C., Tamura, K., (2018). MEGA X: Molecular evolutionary genetics analysis across computing platforms. Mol Biol Evol 35, 1547–1549. doi:10.1093/molbev/msy096

McFadden, C.S., Sánchez, J.A., France, S.C., (2010). Molecular phylogenetic insights into the evolution of Octocorallia: A review. Integr Comp Biol 50, 389–410. doi:10.1093/ICB/ICQ056

McFadden, C.S., Tullis, I.D., Hutchinson, M.B., Winner, K., Sohm, J.A., (2004). Variation in coding (NADH dehydrogenase subunits 2, 3, and 6) and noncoding intergenic spacer regions of the mitochondrial genome in Octocorallia (Cnidaria: Anthozoa). Mar. Biotechnol. 6, 516–526. doi:10.1007/S10126-002-0102-1

Sánchez, J.A., McFadden, C.S., France, S.C., Lasker, H.R., (2003). Molecular phylogenetic analyses of shallow-water Caribbean octocorals. Mar Biol 142, 975–987. doi:10.1007/S00227-003-1018-7

WoRMS Editorial Board, (2024). World Register of Marine Species. https://doi.org/10.14284/170
